# Structure‐Based Macrocyclization of Substrate Analogue NS2B‐NS3 Protease Inhibitors of Zika, West Nile and Dengue viruses

**DOI:** 10.1002/cmdc.202000237

**Published:** 2020-06-30

**Authors:** Niklas J. Braun, Jun P. Quek, Simon Huber, Jenny Kouretova, Dorothee Rogge, Heike Lang‐Henkel, Ezekiel Z. K. Cheong, Bing L. A. Chew, Andreas Heine, Dahai Luo, Torsten Steinmetzer

**Affiliations:** ^1^ Institute of Pharmaceutical Chemistry Philipps University Marbacher Weg 6 35032 Marburg Germany; ^2^ Lee Kong Chian School of Medicine Nanyang Technological University, EMB 03–07 59 Nanyang Drive Singapore 636921 Singapore; ^3^ Institute of Structural Biology Nanyang Technological University EMB 06–01 59 Nanyang Drive Singapore 636921 Singapore; ^4^ School of Biological Sciences Nanyang Technological University 60 Nanyang Dr. Singapore 637551 Singapore; ^5^ Institute of Health Technologies Interdisciplinary Graduate Programme Nanyang Technological University 61 Nanyang Dr. Singapore 637335 Singapore

**Keywords:** dengue virus, drug design, NS2B-NS3 protease, Zika virus, West Nile virus

## Abstract

A series of cyclic active‐site‐directed inhibitors of the NS2B‐NS3 proteases from Zika (ZIKV), West Nile (WNV), and dengue‐4 (DENV4) viruses has been designed. The most potent compounds contain a reversely incorporated d‐lysine residue in the P1 position. Its side chain is connected to the P2 backbone, its α‐amino group is converted into a guanidine to interact with the conserved Asp129 side chain in the S1 pocket, and its C terminus is connected to the P3 residue via different linker segments. The most potent compounds inhibit the ZIKV protease with *K*_i_ values <5 nM. Crystal structures of seven ZIKV protease inhibitor complexes were determined to support the inhibitor design. All the cyclic compounds possess high selectivity against trypsin‐like serine proteases and furin‐like proprotein convertases. Both WNV and DENV4 proteases are inhibited less efficiently. Nonetheless, similar structure‐activity relationships were observed for these enzymes, thus suggesting their potential application as pan‐flaviviral protease inhibitors.

## Graphical abstract

**Cyclic active‐site‐directed inhibitors** of the NS2B‐NS3 proteases from Zika‐, West Nile‐, and dengue‐4 viruses have been developed. The most potent compounds contain a reversely incorporated d‐lysine residue at the P1 position and inhibit the Zika virus protease with low‐nanomolar *K*_i_ values. The crystal structures of seven ZIKV protease inhibitor complexes were determined to support the design of the inhibitors.

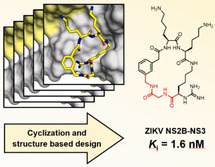

## Introduction

The genus flavivirus comprises nearly 80 members^1^ including numerous human pathogenic viruses such as the mosquito‐borne dengue, West Nile, Zika, Yellow fever, Japanese encephalitis, and St. Louis encephalitis virus, as well as tick‐borne viruses like tick‐borne encephalitis virus of European and Far Eastern subtypes (TBEV‐EU and TBEV‐FE) or Omsk hemorrhagic fever virus. A strong threat is caused by dengue viruses, which are endemic in more than 100 countries. Approximately 40 % of the world's population lives in areas with a risk for dengue virus infections, which can lead to dengue hemorrhagic fever or dengue shock syndrome. A few years ago, the WHO has declared dengue infections as neglected tropical disease. Moreover, after an outbreak in the USA in 1999, the West Nile virus has spread across the USA and Canada. In the case of Zika virus infections, a severe outbreak in South America occurred in 2016.

Flaviviruses are enveloped positive‐strand RNA viruses. Their genome is translated into a single polyprotein that is processed by the own viral protease, the host protease furin and the signal peptidase, thereby producing three structural and several nonstructural proteins. The viral protease consists of the N‐terminal third of the nonstructural protein NS3 containing the catalytic triad composed of residues Ser135, His51, and Asp75. NS3 has to form a complex with its cofactor NS2B to achieve proteolytic activity. The recommended common name of these flavivirus NS2B‐NS3 proteases is flavivirin.^1^ Due to their essential function in the processing of the viral polyprotein, they emerged as potential antiviral drug targets.^2^ The flavivirins from different viruses share a significant sequence homology between 30–55 % in their catalytic NS3 domain and possess similar substrate specificities.^1^ Most natural substrates contain two basic residues in P1 (usually Arg, rarely Lys) and P2 positions (Arg or Lys); very often, a third basic amino acid is found as P3 residue.^3^, ^4^ Therefore, most of the more potent substrate analogue active site‐directed inhibitors of dengue,^5^, ^6^ West Nile,^7^, ^8^, ^9^ and Zika virus^10^, ^11^ contain tribasic or at least dibasic P3‐P1 segments.

**Figure 1:**
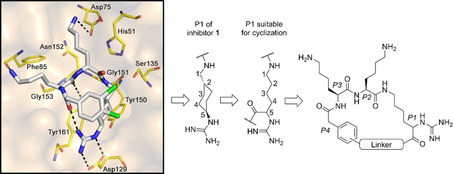
Design strategy for macrocyclic inhibitors starting from the crystal structure of 3,4‐dichloro‐Phac‐Lys‐Lys‐GCMA (**1**) in complex with the WNV protease (left, PDB ID: 2YOL).^12^ Replacement of the P1 *trans*‐(4‐guanidino)cyclohexylmethylamide (GCMA) residue by a reversely incorporated *N* ^*α*^(carbamidoyl)lysine (N(Ca)Lys) enables cyclization to the P4 Phac group via a suitable linker segment.

We have described a related tribasic inhibitor structure of the flavivirin from WNV containing the decarboxylated arginine mimetic *trans*‐(4‐guanidino)cyclohexylmethylamide (GCMA) as P1 residue. It turned out that the incorporation of the GCMA group resulted in a three‐ and sixfold improved potency compared with the analogous P1 agmatine and homoagmatine inhibitors, respectively.^12^ The crystal structure of the noncovalently bound inhibitor 3,4‐dichloro‐Phac‐Lys‐Lys‐GCMA (**1**, *K*_i_=0.13 μM) in complex with the WNV protease (PDB ID: 2YOL) revealed a horseshoe‐like backbone conformation resulting in a close proximity between the P4 phenylacetyl (Phac) residue and the P1 group (Figure 1).^12^ Similar binding modes in the active site of the flavivirin from WNV have been determined for the covalently bound arginal derivatives naphthyl‐2‐carbonyl‐Lys‐Lys‐Arg‐H (PDB ID: 3E90)^13^ and benzoyl‐Nle‐Lys‐Arg‐Arg‐H (PDB ID: 2FP7),^7^ as well as for the latter compound in complex with the dengue‐3 protease (PDB ID: 3U1I).^14^ All these structures suggested to improve the inhibitory potency through macrocyclization between the P1 residue and the N‐terminal acyl group. Due to the open and flat S1 site, we have assumed that it might be sterically possible to replace the GCMA residue by a reversely incorporated lysine or ornithine residue, where its side chain amino group is coupled to the P2 residue and its α‐amino group is converted into a guanidine addressing the Asp129 side chain at the bottom of the flat S1 pocket. Such structures would resemble branched homoagmatine or agmatine analogues containing an additional carboxyl group suitable for a convenient macrocyclization to a P4 phenylacetyl residue with an appropriate amino function, via an appropriate linker segment (Figure 1).

We also speculated that this strategy should provide specific flavivirin inhibitors with negligible inhibitory potencies against trypsin‐like serine proteases or furin‐like proprotein convertases, which also share a preference for basic substrate sequences. These proteases possess very deep S1 pockets, which should not tolerate a branching close to the P1 guanidine at the bottom of their S1 site. Meanwhile, a reliable crystallization protocol using an unlinked binary ZIKV NS2B‐NS3 protease (bZiPro) construct has been established, which provided several structures in complex with various inhibitors.^15^, ^16^, ^17^, ^18^, ^19^ bZiPro is inhibited ten times more strongly by compound **1** (*K*_i_=14 nM) than the WNV protease. Using this bZiPro construct, we obtained a crystal structure in complex with a first macrocyclic inhibitor, which served as a starting point for the design of numerous analogues with improved potencies as found by enzyme kinetic measurements using bZiPro, bD4Pro and the flavivirin from WNV. In addition, we could determine six additional inhibitor structures in complex with bZiPro. These structures contributed to the improvement of the inhibitory potency of these compounds and explained the found structure–activity relationship.

## Results

### Inhibitors containing a P4 4‐aminomethyl‐Phac residue

**Figure 2:**
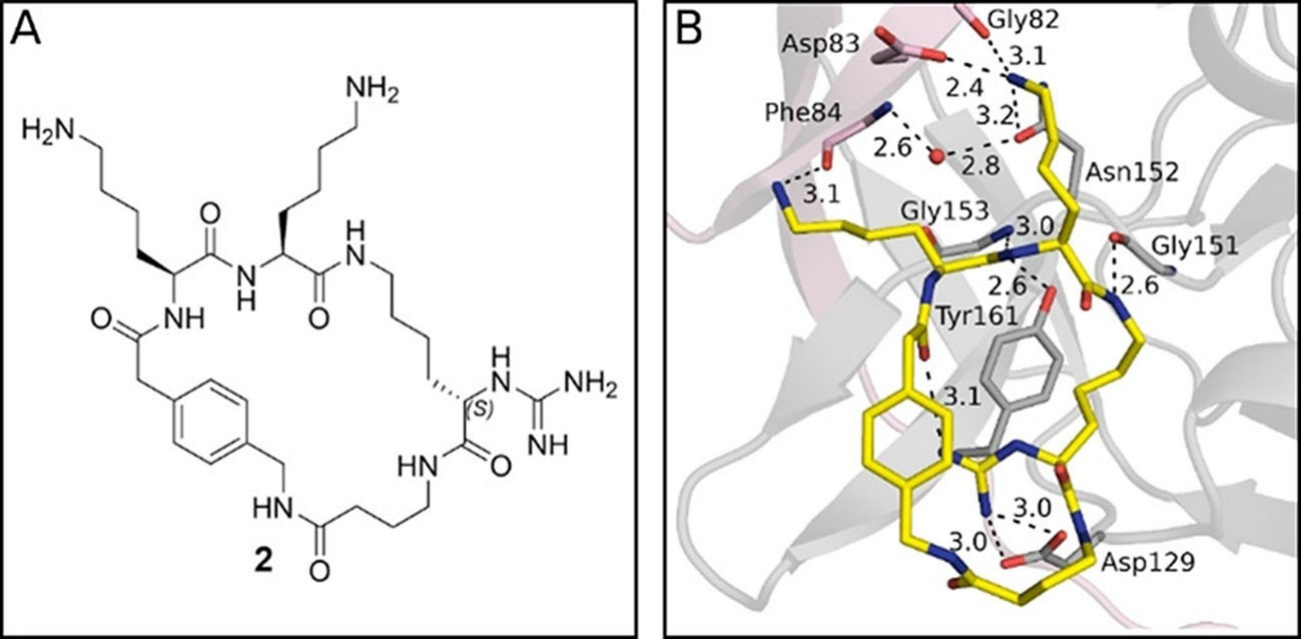
Structure of the macrocyclic inhibitor **2** in complex with bZiPro (PDB ID: 6KK2). A) Chemical structure of the P1 Lys‐derived inhibitor **2**. B) X‐ray structure of bZiPro in complex with inhibitor **2** given as stick model with carbon atoms in yellow. Selected residues of NS2B (pink carbon atoms) and NS3 (gray carbon atoms) involved in polar contacts (dashed black lines, distances in Å) to the inhibitor are labeled and indicated as sticks (for clarity, only the main‐chain or side‐chain atoms involved in contacts are shown). In addition, a water molecule bridging the NS3 residue Asn152 and the Phe84 NH of NS2B is given as a red sphere.

Initially, the macrocyclic P1 Lys‐derived inhibitor **2** and its shortened Orn analogue **3** have been prepared (Table 1). They contain a 4‐aminomethyl substitution on the P4 Phac moiety connected via a γ‐aminobutyric acid (GABA) linker to the P1 residue. Despite weak electron density in the linker segment (Figure S1A in the Supporting Information), a first crystal structure of bZiPro in complex with the cyclic inhibitor **2** could be determined (Figure 2).

**Table 1:** Structures, ring sizes and potencies of the 4‐aminomethyl‐Phac‐derived inhibitors **2**–**7**.

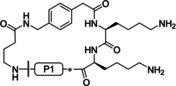					
No.	Ring size	P1	*K*_i_ [μM]^[a]^		
			bZiPro	WNV‐Pro	bD4Pro
**2**	26	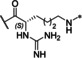	0.0918 ±0.0060	6.30 ±0.49	11.0 ±1.0
**3**	25	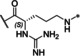	0.846 ±0.111	27.8 ±4.4	117 ±5
**4**	26	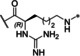	0.0701 ±0.0092	4.97 ±0.50	4.73 ±0.50
**5**	25	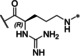	3.61 ±1.33	22.0 ±4.0	60.5 ±5.7
**6**	26	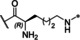	24.7 ±7.2	68.1 ±5.0	94.5 ±11.6
**7**	25	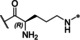	13.2 ±1.7	31.7 ±3.6	30.6 ±3.9

[a] Data are the mean±SD of at least three independent measurements.

The bound structure of inhibitor **2** revealed a suboptimal interaction in the S1 pocket; only one nitrogen of the P1 guanidino group binds to both oxygen atoms of the Asp129 carboxylate with distances of 3.0 Å. The binding mode suggested the preparation of analogous compounds **4** and **5** with *R*‐configuration in P1 position to improve the interaction pattern of the P1 guanidine with Asp129. The amino analogues **6** and **7** have been prepared as reference compounds to find out the contribution of the complete guanidino group to the binding affinity. The poor potency of both amino compounds indicated the importance of the P1 guanidino anchor for binding affinity (Table 1).

**Figure 3:**
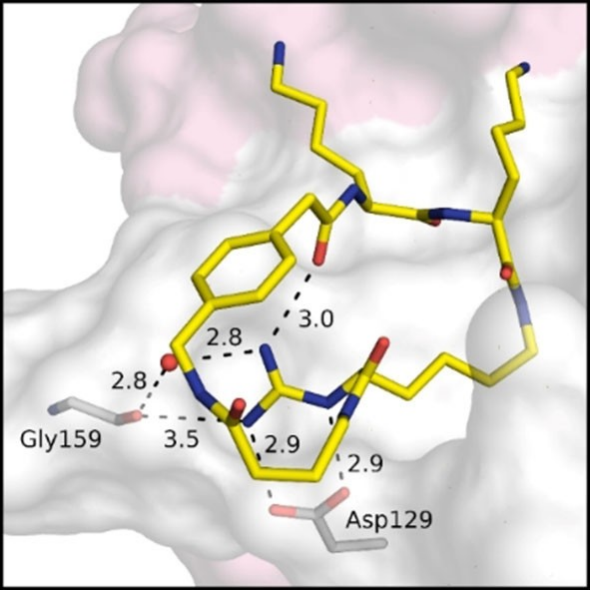
Structure of bZiPro (NS2B shown with transparent surface in pink, NS3 with transparent surface in gray) in complex with the cyclic inhibitor **4** (stick model with carbon atoms in yellow, PDB ID: 6KK4). The incorporation of an *R*‐configured P1 residue enabled the formation of an improved interaction of the P1 guanidine to Asp129 (distances in Å).

For the most potent inhibitor **4** of this series, a crystal structure in complex with bZiPro has been determined (Figure 3). The structure revealed an improved dual interaction pattern of the P1 guanidino group with Asp129 and two additional contacts to the carbonyl oxygen of Gly159. A direct, but weak hydrogen bond (3.5 Å) is formed between Gly159 and the guanidino group and a second interaction via a bridging water molecule (Figure 3). An intramolecular H‐bond between the P1 guanidine and the P4 carbonyl oxygen further stabilizes the inhibitor conformation. The P4 Phac residue is placed like a hydrophobic shield above the salt bridge to Asp129 and may strengthen the electrostatic contacts by displacing competing water molecules. Moreover, the relatively long and solvent‐exposed linker segment suggested to use the *meta*‐position on the Phac residue for cyclization and to truncate the linker part leading to more constrained structures.

### Inhibitors with P4 *meta*‐substituted Phac residues

The incorporation of *meta*‐substituted Phac residues provided inhibitors **8**–**19** (Table 2). A more than tenfold improved potency against bZiPro was found for inhibitor **8**, the direct analogue of the *para*‐substituted compound **4**. Despite slightly reduced effects, a stronger inhibition was also found for the WNV and DENV4 proteases. Stepwise truncation of the linker segment by replacing GABA with β‐Ala or Gly provided the further improved analogues **9** and **10**. Compound **10** was the best inhibitor of this series exhibiting *K*_i_ values of 1.6, 138, and 142 nM against the flavivirins from ZIKV, WNV and DENV4, respectively.

**Table 2:** Structures and potencies of inhibitors **8**–**19** possessing a *meta*‐substituted Phac‐residue in P4 position.

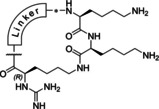					
No.	Ring size	Linker	*K*_i_ [μM]^[a]^		
			bZiPro	WNV‐Pro	bD4Pro
**8**	25	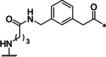	0.00433 ±0.00035	0.702 ±0.083	0.286 ±0.009
**9**	24	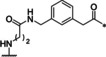	0.00388 ±0.00081	0.374 ±0.044	0.182 ±0.005
**10**	23	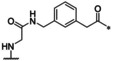	0.00157 ±0.00032	0.138 ±0.031	0.142 ±0.010
**11**	22	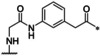	1.42 ±0.27	47.6 ±2.5	60.6 ±5.3
**12**	20	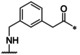	0.0373 ±0.0047	15.3 ±0.8	1.28 ±0.05
**13**	23	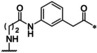	0.0802 ±0.0097	4.28 ±0.31	7.96 ±0.55
**14**	24	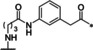	0.106 ±0.029	16.5 ±4.6	5.80 ±0.36
**15**	23	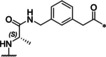	0.0182 ±0.0051	1.39 ±0.32	3.60 ±0.38
**16**	23	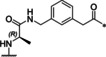	0.00352 ±0.00109	0.237 ±0.021	0.125 ±0.004
**17**	23	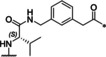	0.0428 ±0.0022	6.71 ±0.32	2.81 ±0.18
**18**	23	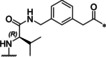	0.00774 ±0.00066	0.439 ±0.028	0.486 ±0.027
**19**	23	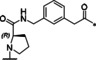	0.0177 ±0.0005	0.850 ±0.068	4.08 ±0.26

[a] Data are the mean±SD of at least three independent measurements.

**Figure 4:**
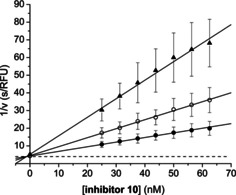
Dixon plot of the bZiPro (1.96 nM in assay)‐catalyzed cleavage of the substrate Phac‐Leu‐Lys‐Lys‐Arg‐AMC (• 10 μM, ○ 5 μM, and ▴ 2.5 μM) in the presence of inhibitor **10**. The dashed line represents 1/*V*_max_.

Further truncation of the linker by replacement of 3‐aminomethyl‐Phac with 3‐amino‐Phac in combination with Gly (**11**) resulted in a drastic drop of the inhibitory potency against all tested enzymes. We speculated to regain potency by coupling the longer β‐Ala or GABA instead of Gly to the 3‐amino‐Phac residue. However, kinetic measurements revealed only a minor beneficial effect for inhibitors **13** and **14**. This suggests that the inhibitory potency not only depends on the number of linker atoms but also on the appropriate position of the peptide bond between the Phac residue and the next amino acid. To our surprise, a relatively strong potency (*K*_i_=38 nM) against bZiPro was found for the shortest analogue **12**, where the 3‐aminomethyl‐Phac residue was directly coupled to the P1 carboxyl group without any bridging residue. Otherwise, the same compound is only a weak inhibitor of the WNV and DENV4 proteases. Figure 4 shows the Dixon plot^20^ of the bZiPro inhibition by inhibitor **10** indicating the competitive reversible binding mode.

**Figure 5:**
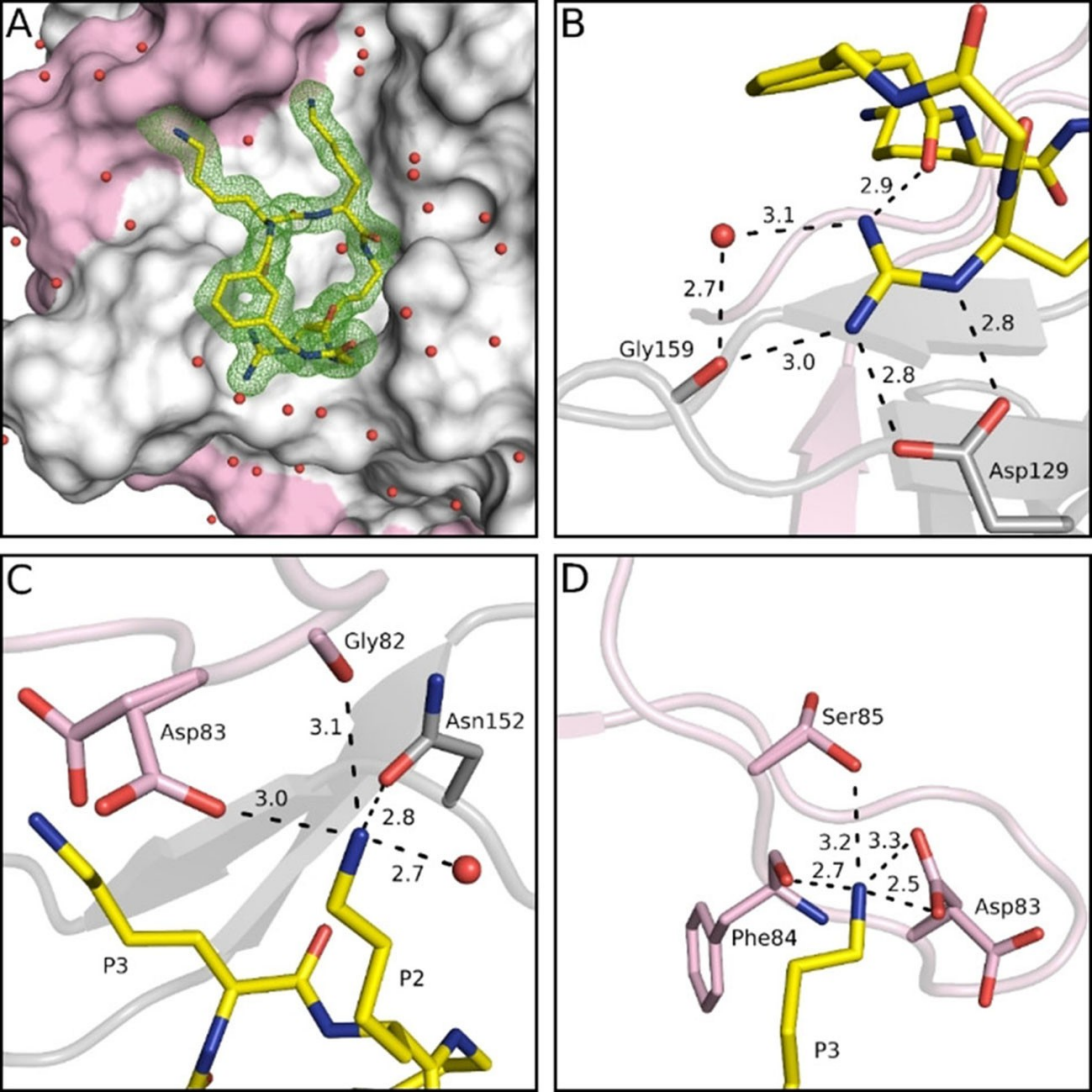
Crystal structure of bZiPro in complex with inhibitor **10** (PDB ID: 6Y3B), shown as stick model with carbon atoms in yellow, oxygen in red, and nitrogen in blue. Water molecules are shown as red spheres, and polar contacts as black dashed lines with distances in Å. Residues of NS2B and NS3 are given with pink and gray carbon atoms, respectively. A) Surface representation of bZiPro in complex with inhibitor **10**, shown with its electron density difference map (*F*_o_−*F*_c_) as green mesh at the 3*σ* level. B) Interactions of the P1 guanidine at the bottom of the S1 pocket, C) polar contacts of the P2 Lys side chain with residues in the S2 pocket, and D) interactions of the P3 Lys side chain. The NS2B residues Asp83 and Ser85 exist in two conformations.

A crystal structure was determined for the best inhibitor **10** in complex with bZiPro (Figure 5). The P1 interactions are similar to those described above for the complex with inhibitor **4**, although a stronger contact (3.0 Å) between the P1 guanidine and Gly159 is formed (Figure 5B). Interestingly, two conformations of the side chain from NS2B residues Asp83 and Ser85 were found. In one conformation, the side chain of Asp83 is directed to the P2 Lys amino group (occupancy 53 %) as found in most other complexes (Figure 5C). In the second conformation it interacts with the P3 Lys amino group (occupancy 47 %, Figure 5D). In case of Ser85, approximately 37 % of its side chain is directed to the P3 Lys residue, whereas the major part (63 %) points upwards lacking any contact to the ligand. The P2 and P3 backbone interactions are identical to those shown above for the complex with inhibitor **2** (Figure S2).

**Figure 6:**
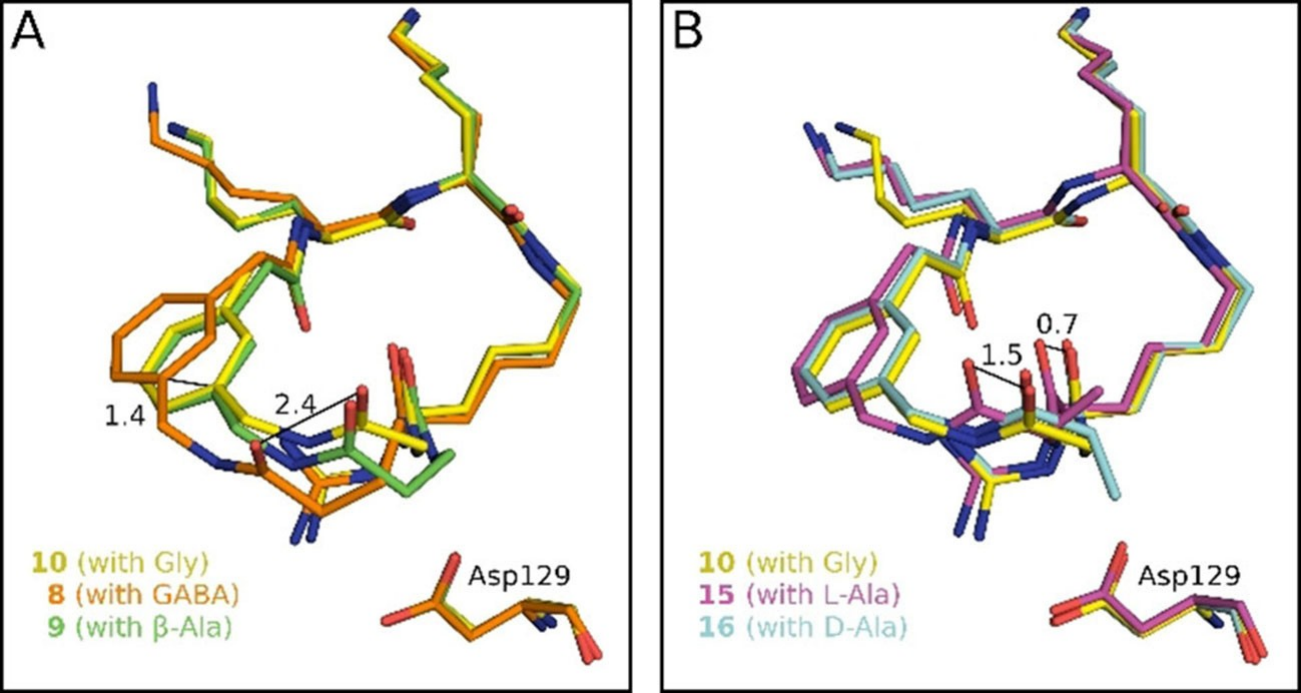
A) Superimposition of the bound conformations of Gly inhibitor **10** (carbon atoms in yellow, PDB ID: 6Y3B) with the elongated GABA derivative **8** (carbon atoms in orange, PDB ID: 6KPQ) and the β‐Ala compound **9** (carbon atoms in green, PDB ID: 6KK4) taken from complexes with bZiPro. An identical backbone conformation was observed for the P3–P1 segment of all three inhibitors, whereas the backbone of the linker between the P1 and P3 residues and the position of the phenyl ring is slightly shifted between these analogues. Distances are shown as lines and given in Å. B) Superimposition of the bound conformations of the Gly inhibitor **10** (carbons in yellow) with the Ala compound **15** (carbons in magenta, PDB ID: 6KK5) and the dAla derivative **16** (carbons in light blue, PDB ID: 6KK6). Identical backbone conformations for the segment between the Phac and the P1 residue were determined for the more potent Gly and dAla inhibitors, which is shifted for the less effective Ala analogue.

Additional crystal structures were determined for bZiPro complexes with the elongated inhibitors **8** and **9**. The insertion of one or two methylene groups leads to a widening of the backbone from the linker segment. In the case of the GABA‐derived inhibitor **8**, it comes to a 1.4 Å displacement of the P4 phenyl ring compared with the structure of analogue **10**. Otherwise, the P1 guanidino groups adopt an identical position in all three structures (Figure 6A). Furthermore, the replacement of Gly in inhibitor **10** by Ala or dAla as well as by Val and dVal (**15**–**18**) was tested. In case of both pairs, the incorporation of an *R*‐configured linker residue was strongly preferred, although these analogues and the dPro derivative **19** were less potent than inhibitor **10**. The crystal structure analysis of bZiPro in complex with the Ala and dAla inhibitors **15** and **16** revealed that the side chain of this linker residue points toward the solvent and is not involved in contacts to bZiPro. The P1 interactions to Asp129 and the binding pattern of the P2 and P3 side chains are identical to inhibitor **10**. In contrast, the incorporation of Ala induced a slightly different backbone conformation of the linker segment leading to a shift of the carbonyl from the amide bond to the 3‐aminomethyl‐Phac residue by 1.5 Å (Figure 6B). This displacement also leads to a slight shift of the P4 aryl ring. These changes contribute to the significantly reduced potency of the Ala inhibitor **15** compared with analogues **10** and **16**.

For comparison, two additional analogues of the most potent compound **10** have been prepared (structures shown below Table S1). As with inhibitor **6**, in derivative **20** the P1 amino group was not converted into a guanidine moiety, which drastically reduced the potency towards ZIKV, WNV, and DENV4 proteases to *K*_i_ values of 23, 99, and 282 μM respectively. A similar effect was observed by conversion of the P1 amino group into a ureido moiety (−NHCONH_2_) in compound **21** leading to inhibition constants of 12, 68, and 135 μM against ZIKV, WNV, and DENV4 proteases respectively.

### Inhibitors with linear linker segment

The 3‐aminomethyl‐Phac residue is contributing with seven atoms to the backbone of the macrocycle. Its replacement with linear amino acids provided compounds **22**–**24** (Table 3). The weakest potency was found for inhibitor **22** containing a five atoms long GABA residue, which appears to be too short for an appropriate cyclization. The stepwise elongation by the incorporation of 5‐aminovaleric acid or 6‐aminocaproic acid provided a significant improvement, albeit the potency of inhibitors **23** and **24** against bZiPro is reduced approximately tenfold compared with the more rigid compound **10**. Therefore, the aliphatic amino acid was replaced by various combinations of Gly and β‐Ala residues to stabilize the inhibitor conformation through an additional peptide bond in case of analogues **25**–**28**. The strongest potency was found for inhibitor **26**. When compared with inhibitor **10**, it possesses a comparable potency for bZiPro and even a slightly enhanced affinity for the WNV flavivirin.

**Table 3:** Structures and potencies of inhibitors **22**–**28** possessing aliphatic amino acids in the linker segment.

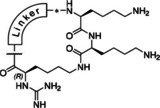					
No.	Ring size	Linker	*K*_i_ [μM]^[a]^		
			bZiPro	WNV‐Pro	bD4Pro
**22**	21	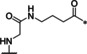	0.124 ±0.008	6.70 ±0.52	6.09 ±0.47
**23**	22	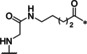	0.0167 ±0.0034	1.22 ±0.02	1.12 ±0.05
**24**	23	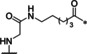	0.0154 ±0.0026	0.973 ±0.173	1.99 ±0.09
**25**	22	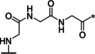	0.00374 ±0.00020	0.129 ±0.007	0.378 ±0.027
**26**	23	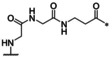	0.00172 ±0.00003	0.0686 ±0.0131	0.160 ±0.005
**27**	23	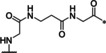	0.00735 ±0.00039	0.468 ±0.003	0.815 ±0.079
**28**	23	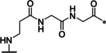	0.0128 ±0.0005	0.974 ±0.134	2.72 ±0.19

[a] Data are the mean±SD of at least three independent measurements.

### Linear reference inhibitors

To investigate the influence of the cyclization on inhibitory efficacy, four linear reference compounds have been prepared (Table 4). Compound **29**, lacking the peptide bond between the P1 carboxyl group and the Gly residue, is a more than 1000 times less potent inhibitor of bZiPro than analogue **10** and a very weak inhibitor of the WNV and DENV proteases. We speculated that its reduced affinity is not only caused by the increased conformational flexibility, but also by the disturbing charge of the free P1 carboxyl group, which has to be accommodated close to Asp129 in the S1 site. This assumption was confirmed by the more than 100‐fold improved potency of the uncharged amide analogue **30**, although it still exhibits a tenfold reduced potency compared with compound **10**. A similar potency was found for the acetylated reference compound **31**, which lacks the positively charged amino group present on the Gly residue of inhibitor **30**. Furthermore, the complete deletion of the −NH−CO− segment in inhibitor **10** provided the homoagmatine derivative **32**. This compound is significantly less potent than analogues **30** and **31**, probably caused by the increased flexibility of the homoagmatine group in solution, which would lead to a stronger entropic penalty during binding to the protease.

**Table 4:** Structures and potencies of linear reference inhibitors **29**–**32**.

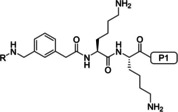					
No.	R	P1	*K*_i_ [μM]^[a]^		
			bZiPro	WNV‐Pro	bD4Pro
**29**	H−Gly	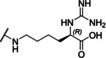	2.04 ±0.60	51.6 ±0.7	75.0 ±3.4
**30**	H−Gly	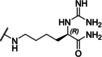	0.0136 ±0.0026	1.55 ±0.38	0.775 ±0.047
**31**	Ac	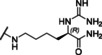	0.00948 ±0.00061	1.48 ±0.10	1.21 ±0.09
**32**	Ac	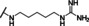	0.0882 ±0.0038	4.94 ±0.53	18.1 ±1.2

[a] Data are the mean±SD obtained from at least three independent measurements.

### Correlation of the inhibitory potencies between the used proteases

**Figure 7:**
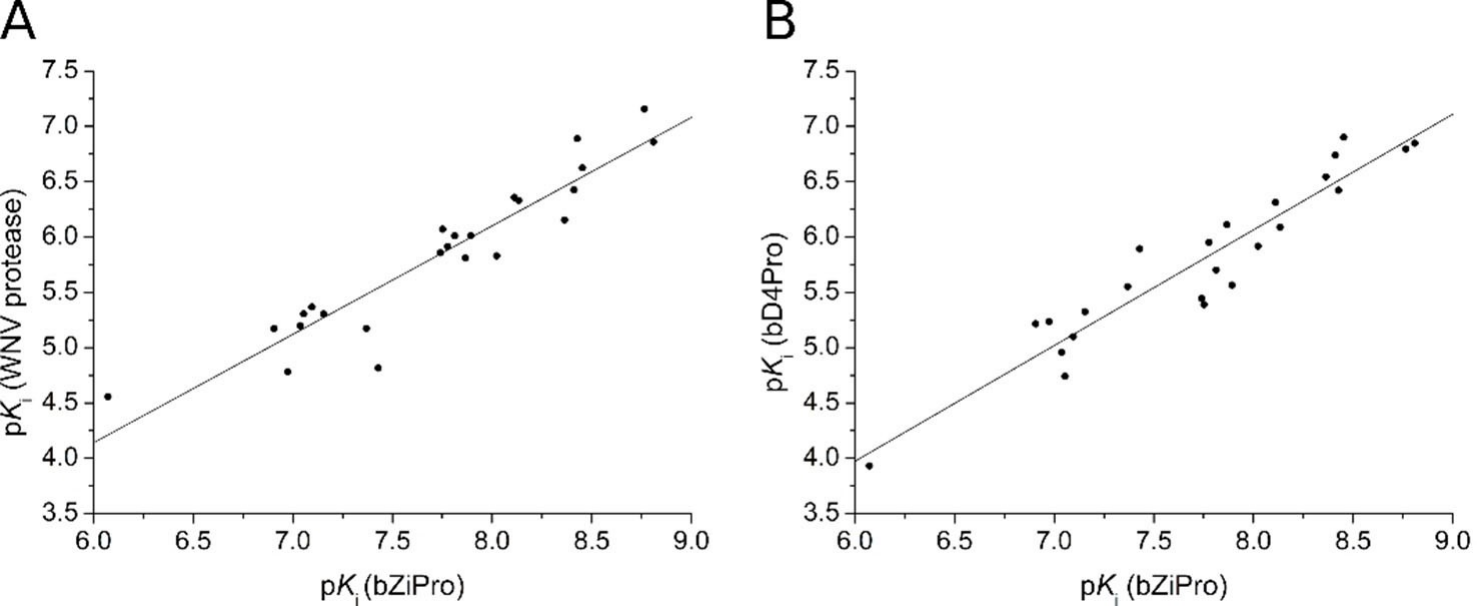
Correlation of the determined p*K*_i_ values for the inhibition of bZiPro and A) the WNV protease or B) the bD4Pro for all inhibitors with inhibition constants <1 μM against bZiPro (*n*=24). For the bZiPro/WNV protease correlation, a slope of 0.98 and an intercept of −1.74 were calculated, for bZiPro/bD4Pro a slope of 1.05 and an intercept of −2.30. The slope close to 1 reveals a similar SAR for both correlations. Based on the intercepts, an approximately 55‐fold (10^1.74^) or 200‐fold (10^2.30^) stronger inhibition of bZiPro compared with the WNV protease and bD4Pro, respectively, can be calculated on average.

With few exceptions, the more potent inhibitors with *K*_i_ values <1 μM against bZiPro are approximately 50 and 200 times less efficient against the WNV protease and bD4Pro respectively. The different potencies might be caused by the influence of the NS2B residue 83, which is Asp in bZiPro that corresponds to Asn84 in the WNV protease and Thr or Ser in position 84 of the dengue proteases of serotypes 1–4. In case of the ZIKV protease, it was previously suggested that Asp83 contributes to stronger contacts with substrate‐analogue inhibitors containing Lys or Arg as P2 residue.^10^ Despite significant differences in the absolute *K*_i_ values, there is a similar tendency of the potency ranking between bZiPro and the WNV or DENV enzymes (Figure 7). This trend can be explained by the rather conserved active site architecture among the flavivirins, which should allow the development of pan‐flavivirus protease inhibitors based on the present macrocyclic inhibitor type.

### Synthesis

**Scheme 1:**
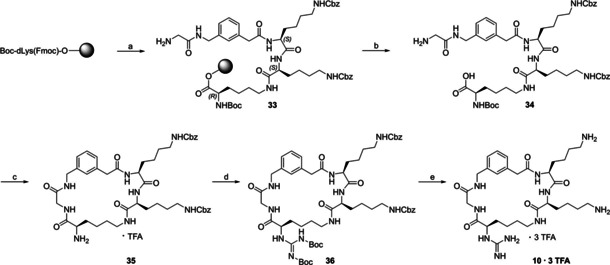
Synthesis of inhibitor **10** on 2‐CTC‐resin loaded with Boc‐dLys(Fmoc)‐OH. a) Standard Fmoc SPPS, subsequent coupling of 2×Fmoc‐Lys(Cbz)‐OH, Fmoc‐3‐aminomethyl‐Phac‐OH, and Fmoc‐Gly‐OH using a fourfold excess of standard Fmoc‐amino acids, HBTU and eightfold excess of DIPEA, whereas only a 2.5‐fold excess of Fmoc‐3‐aminomethyl‐Phac‐OH in the presence of 2.5 equiv. HBTU and 5 equiv. DIPEA was used; Fmoc cleavage with 20 % piperidine in DMF (5 and 15 min); b) 1 % TFA in CH_2_Cl_2_ (4×30 min, RT), neutralization with DIPEA; c) i) 3 equiv. HATU and 6 equiv. DIPEA, DMF, 3 h, RT, ii) 4 N HCl in dioxane, 2 h, RT, preparative HPLC; d) 4.5 equiv. of *N,N’*‐di‐Boc‐1*H*‐pyrazole‐1‐carboxamidine, 7.5 equiv. of DIPEA, DMF, 48 h; e) 33 % HBr in acetic acid, precipitation in diethyl ether, preparative HPLC.

The synthesis of the cyclic inhibitors was achieved by a combination of solid phase peptide synthesis (SPPS) and subsequent solution synthesis, as depicted in (Scheme 1) for the most potent bZiPro inhibitor **10**. For SPPS, a standard Fmoc strategy on 2‐chloro‐tritylchloride resin was used. The resin was initially loaded with Boc‐dLys(Fmoc)‐OH. After removal of the Fmoc protection, the dLys side chain was subsequently coupled with the P2 and P3 residues (2×Fmoc‐Lys(Cbz)‐OH), the P4 Fmoc‐3‐aminomethyl‐Phac‐OH residue and Fmoc‐Gly‐OH as linker segment. After each coupling step, Fmoc was removed by 20 % piperidine in DMF. The resin‐bound intermediate **33** was treated with 1 % TFA in CH_2_Cl_2_ providing the side chain protected crude intermediate **34**, which was cyclized using 3 equiv. HATU and 6 equiv. DIPEA in DMF (peptide concentration≈2 mM).^21^ The crude product was treated with 4 N HCl in dioxane providing intermediate **35**, which was purified by preparative reversed‐phase HPLC. The amino group was converted into a bis‐Boc‐protected guanidine^22^ yielding intermediate **36**, which was deprotected by 33 % HBr in acetic acid. The inhibitor was finally purified by preparative HPLC.

The cyclic analogues **2**–**5** (Table 1), **8**–**19** (Table 2) and **22**–**28** (Table 3) were prepared by an identical strategy starting from 2‐CTC‐resin loaded with the required Nα‐Boc‐ and side‐chain Fmoc‐protected Lys or Orn derivative in the required configuration. In some cases the P4 Fmoc‐3‐aminomethyl‐Phac‐OH residue was replaced by either Fmoc‐4‐aminomethyl‐Phac‐OH, Fmoc‐3‐amino‐Phac‐OH, FmocGABA‐OH, Fmoc‐5‐Ava‐OH, Fmoc‐6‐Ahx‐OH, Fmoc‐Gly‐OH, or Fmoc‐β‐Ala‐OH. Beside Fmoc‐Gly‐OH, also Fmoc‐β‐Ala‐OH, Fmoc‐γ‐GABA‐OH, Fmoc‐Ala‐OH, Fmoc‐dAla‐OH, Fmoc‐Val‐OH, Fmoc‐dVal‐OH or Fmoc‐dPro‐OH were used for the incorporation of the linker segment.

For inhibitors **6**, **7**, and **20** containing a free P1 amino group, the intermediate obtained after the cyclization step and removal of the Boc‐protecting group was purified by preparative HPLC, followed by Cbz‐deprotection with 33 % HBr in acetic acid and subsequent purification by HPLC. In case of the urea derivative **21**, the purified intermediate **35** was treated with trimethylsilylisocyanate,^23^ followed by removal of the protecting groups with 33 % HBr in acetic acid. So far, we have started the syntheses of all cyclic compounds with the resin loading through the P1 residue. The Nα‐Boc‐protection of intermediate **34** (Scheme 1) enables a racemization‐free cyclization to glycine or other residues. In principle, the syntheses could be alternatively started with all other residues, as long as a racemization free cyclization is possible.

The linear inhibitor **29** was also prepared on Boc‐dLys(Fmoc)‐2‐CTC resin, with Cbz‐Gly‐OH coupled as final residue in the SPPS. After mild acidic cleavage from resin and Boc deprotection with 4 N HCl/dioxane, the amine was converted into a bis‐Boc‐protected guanidine as described above, followed by final deprotection with 33 % HBr in acetic acid. The amide analogues **30** and **31** were analogueously prepared on Fmoc‐Sieber‐amide‐resin by coupling of Cbz‐Gly‐OH or acetic acid to the 3‐aminomethyl‐Phac‐residue on the solid support. The following steps were performed as described for compound **29**. The synthesis of the homoagmatine inhibitor **32** started from tritylchloride resin, which was loaded with cadaverine as described previously.^12^ After mild acidic cleavage from resin, the amino group was converted into a guanidine, followed by treatment with 33 % HBr in acetic acid. The analytical data of the final inhibitors are provided in the Supporting Information.

## Discussion

Most of the already known and more effective flavivirin inhibitors^2^, ^24^ belong either to the group of allosteric small molecule,^25^, ^26^, ^27^, ^28^ allosteric macrocyclic^29^ or active site‐directed, reversible competitive inhibitors. Some of the latter group, like the peptidic arginal^8^ or boroarginine derivatives,^10^ form a reversible covalent bond with their target. In case of the activated pyrazole esters^17^ a relatively stable acyl‐enzyme is formed, whereas other inhibitors bind without covalent interaction,^9^, ^12^, ^30^ but are usually less potent. In principle, most small molecule allosteric ligands possess more promising drug‐like properties than multibasic peptidic active site inhibitors. However, studies with HIV and HCV protease inhibitors revealed a stronger tendency for resistance developments against allosteric ligands than against active site inhibitors. With respect to virus replication, mutations in allosteric binding pockets are better tolerated than changes in the active site, which often lead to a more severe influence on the fitness of the virus.^31^, ^32^ This advantage justifies further efforts for the improvement of active site inhibitors. One strategy to reduce the conformational freedom of peptide derivatives and predisposing them to a favorable orientation for binding is their macrocyclization, leading to improved potency, selectivity and stability.^33^ By this approach, we have prepared numerous noncovalent inhibitors with excellent selectivity for the tested flavivirins, when compared with trypsin‐like serine proteases or basic proprotein convertases. Using trypsin and furin as examples, we could not detect any inhibition with compounds **10** and **11** at concentrations of 75 μM in an enzyme kinetic assay. The deep S1 pocket of trypsin^34^ and furin^35^ cannot tolerate a branching close to the basic P1 guanidine anchor, which is well accepted by the flavivirins due to their flat and open S1 site. In contrast to some of our previously described linear C‐terminal elongated derivatives,^18^, ^36^ the cyclic derivatives are fully stable when incubated with bZiPro, as analyzed by mass spectrometry (Figure S3). The crystal structures of bZiPro with all inhibitors containing a d‐configured P1 residue revealed a preferred interaction of the dN(Ca)Lys guanidine to Asp129, enabling an additional contact to the carbonyl of Gly159. This binding pattern to Asp129 seems to be even better suited than the interactions found for various arginal‐derived inhibitors in complex with the WNV protease (PDB ID: 3E90),^13^ the DENV protease of serotype 3 (PDB ID: 3U1I), or bZiPro (PDB ID: 5H6V).^16^ Only one of the terminal guanidine nitrogens binds to Asp129 in these arginal complexes, although an additional polar contact to the carbonyl of the NS3 residue 130 (Phe or Tyr) is formed.

The inhibition constants of analogues **8**–**11** revealed that a 23‐membered macrocycle represents an optimal ring size for targeting all three tested flavivirins and that it comes to a drastic drop in potency after further truncation in case of inhibitor **11**. Surprisingly, with a *K*_i_ value of 37 nM against bZiPro, the two atoms shorter analogue **12** exhibits a stronger potency than compound **11**. The relatively weak inhibition constant of 80 nM against bZiPro for compound **13**, which has the same ring size as the best inhibitor **10**, also suggests a disturbing effect when the amide bond is directly attached to the Phac residue lacking the methylene insertion. Interestingly, in the cyclic series without the Phac residue (**22**–**28**, Table 3) the strongest potency was also found for the 23‐membered inhibitor **26**, although the truncation by one methylene group in case of inhibitor **25** was still tolerated. A significant influence of the amide bond position in the connecting linker segment on inhibitory potency was observed also in this series. This can be seen by comparing compounds **26** and **28**, which both possess 23 ring atoms.

All backbone and side chain interactions of the P2 and P3 residues are very similar to those previously observed for our linear substrate‐analogue inhibitors.^18^ Although we used identical P2 and P3 residues (Lys) in all inhibitors, we have only found two conformations of the Asp83 side chain in the bZiPro/**10** and bZiPro/**9** complexes, which were equally directed toward the S2 or S3 pocket. This flexibility might be relevant for the efficient binding and processing of the natural occurring NS4A/2K cleavage site segment within the ZIKV polyprotein. It contains a P4‐P4′ EKQR↓SPQD sequence with a nonbasic P2 residue, whereas all other natural cleavage sites possess an Arg or Lys in P2 position.^4^ This suggests an improved binding of the NS4A/2K substrate sequence, when the Asp83 side chain is oriented toward the S3 pocket. Interestingly, the experimentally found two orientations of the Asp83 side chain in complexes with inhibitors **10** and **9** were recently predicted based on modeling results with the ZIKV protease in complex with a small molecule inhibitor.^37^

**Figure 8:**
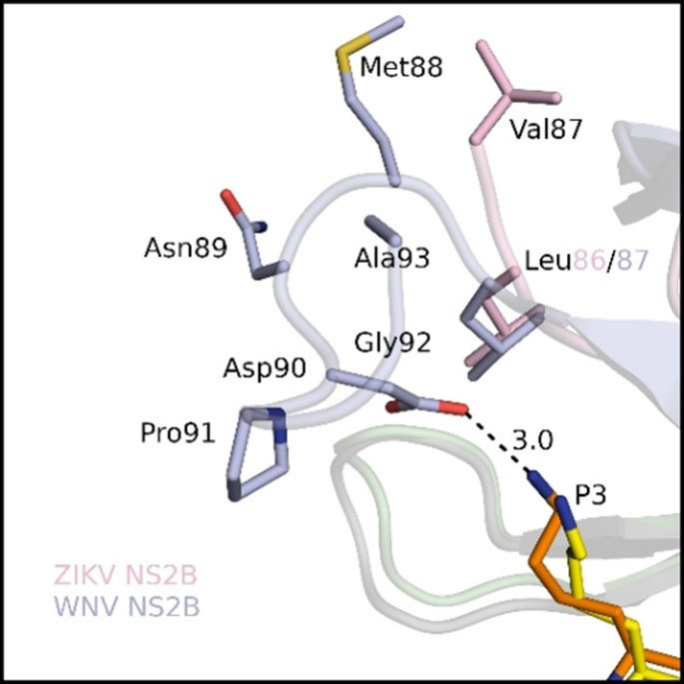
Superimposition of the bZiPro/inhibitor **10** complex (PDB ID: 6Y3B) with the arginal‐derived inhibitor naphthoyl‐Lys‐Lys‐Arg‐H in complex with a covalently connected WNV NS2B‐NS3 construct (PDB ID: 3E90^13^) indicating the influence of the C‐terminal NS2B segment on the formation of the S3 pocket. The visible C‐terminal NS2B segments of bZiPro and the WNV protease are shown in cartoon style with side chains in pink and light blue, respectively (NS3 given as gray or light green cartoon). The P3 side chain of inhibitor **10** is provided with yellow carbon atoms and the arginal derivative with orange carbons.

Despite different crystal forms, electron density is missing after Val87 in all NS2B structures of bZiPro complexes,^15^, ^17^, ^18^ although the NS2B construct ends at Glu96 (C‐terminal segment: V^87^EEDGPPMRE^96^). In a related structure of a covalently connected NS2B‐NS3 construct from WNV in complex with an arginal derived inhibitor, the C‐terminal NS2B segment adopts a loop conformation, which enables an additional contact of its Asp90 side chain with the P3 Lys amino group of the inhibitor (3.0 Å, Figure 8). Assuming a similar C‐terminal backbone conformation, an analogue interaction could also exist in bZiPro complexes in solution via Glu89, which corresponds to Asp90 in NS2B of WNV. However, in both space groups of the obtained bZiPro crystals, an analogue loop conformation is impossible due to crystal packing, leading to a steric clash with residues of a neighboring NS3 molecule.

## Conclusion

Although we could not demonstrate an intracellular antiviral efficacy for these inhibitors, the convenient synthetic access to this type of cyclic compounds in combination with a well‐established structure determination of the bZiPro/inhibitor‐complexes, provides a promising starting point for the development of pan‐flaviviral NS2B‐NS3 protease inhibitors.

## Experimental Section

**General**. Solvents and standard reagents were purchased from Sigma‐Aldrich, Alfa Aesar, Roth, or Merck and used without further purification. Protected Fmoc‐amino acids, coupling reagents, and resins for peptide synthesis were obtained from IRIS Biotech (Marktredwitz, Germany), Orpegen Peptide Chemicals GmbH (Heidelberg, Germany), ChemPur Feinchemikalien und Forschungsbedarf GmbH (Karlsruhe, Germany), and Bachem AG (Bubendorf, Switzerland).

Analytical HPLC experiments were performed on a Primaide (VWR, Hitachi) system (column: NUCLEODUR C_18_ ec, 5 μm, 100 Å, 4.6 mm×250 mm, Macherey‐Nagel). Water (solvent A) and acetonitrile (solvent B), both containing 0.1 % TFA, were used as eluents with a linear gradient (increase of 1 % solvent B/min) and a flow rate of 1 mL/min. The detection was performed at 220 nm. The final inhibitors were purified via preparative HPLC (pumps, Varian PrepStar model 218 gradient system; detector, ProStar model 320; fraction collector, Varian model 701) using a reversed phase column (NUCLEODUR C_18_ ec, 5 μm, 100 Å, 32 mm×250 mm, Macherey‐Nagel) with identical solvents as described for the analytical HPLC and a linear gradient (indicated below) at a flow rate of 20 mL/min (detection at 220 nm). All final inhibitors possess a purity >95 % based on HPLC detection at 220 nm. The inhibitors were obtained as TFA salts after lyophilization on a freeze‐drier (Martin Christ Gefriertrocknungsanlagen GmbH, Osterode am Harz, Germany). The molecular mass of the synthesized compounds was determined using a QTrap 2000 ESI spectrometer (Applied Biosystems).

**Peptide synthesis**. All cyclic peptides and the linear peptide **29** were prepared on 2‐CTC‐resin, which was either loaded with Boc‐dLys(Fmoc)‐OH, Boc‐Lys(Fmoc)‐OH, Boc‐dOrn(Fmoc)‐OH, or Boc‐Orn(Fmoc)‐OH. The linear amide analogues **30** and **31** were synthesized on Fmoc‐Sieber‐amide‐resin, the linear homoagmatine inhibitor **32** was prepared on tritylchloride‐resin, which was initially loaded with cadaverine. The following residues were coupled by manual Fmoc‐SPPS in polypropylene syringes (MultiSynTech, Witten, Germany) or 20 ml glass vessels with filter frits. For coupling of Fmoc‐Lys(Cbz)‐OH, Fmoc‐Gly‐OH, Fmoc‐Ala‐OH, Fmoc‐dAla‐OH, Fmoc‐β‐Ala‐OH, Fmoc‐GABA‐OH, Fmoc‐Val‐OH, Fmoc‐dVal‐OH, Fmoc‐dPro‐OH, Fmoc‐5‐Ava‐OH, Fmoc‐6‐Ahx‐OH, Cbz‐Gly‐OH, and acetic acid, a fourfold excess of the amino acid, HBTU and HOBt in presence of a eightfold excess of DIPEA was used. Fmoc‐3‐aminomethyl‐Phac‐OH, Fmoc‐4‐aminomethyl‐Phac‐OH and Fmoc‐3‐amino‐Phac‐OH were coupled with 2.5 equiv. amino acid, 2.5 equiv. HBTU, 2.5 equiv. HOBt and 5.0 equiv. DIPEA. The synthesis of the most potent cyclic inhibitor **10** is described in the following paragraph in detail.

### 1‐((8*R*,15*S*,18*S*)‐15,18‐bis(4‐aminobutyl)‐4,7,14,17,20‐pentaoxo‐3,6,13,16,19‐pentaaza‐1(1,3)‐benzenacyclohenicosaphane‐8‐yl)guanidine⋅3 TFA (inhibitor 10)

Intermediate **34**. Boc‐dLys(Fmoc)‐OH (868 mg, 1.85 mmol, 1.0 equiv) and DIPEA (1.3 mL, 7.4 mmol, 4.0 equiv) were dissolved in dry CH_2_Cl_2_ (12 mL). The solution was added to 2‐chloro‐tritylchloride resin (1.16 g, 1.0 equiv, resin loading capacity 1.6 mmol/g) in a 20 mL glass reaction vessel equipped with a filter frit. After shaking the mixture for 2 h and removal of the solvent, the resin was treated with CH_2_Cl_2_/MeOH/DIPEA (17 : 2 : 1, *v*/*v*/*v*, 12 mL, 3×1 min), washed with CH_2_Cl_2_ (2×), DMF (3×) and CH_2_Cl_2_ (4×), and dried *in vacuo*. The obtained Boc‐dLys(Fmoc)‐2‐CTC‐resin (1.90 g, loading 0.90 mmol/g) was washed twice with DMF. The Fmoc group was removed by treatment with 20 % piperidine in DMF (5 min and 15 min). After washing with DMF (7×), the resin was treated with a solution of Fmoc‐Lys(Cbz)‐OH (3.44 g, 6.84 mmol, 4.0 equiv), HBTU (2.60 g, 6.84 mmol, 4.0 equiv), HOBt (924 mg, 6.84 mmol, 4.0 equiv) and DIPEA (2.4 mL, 13.7 mmol, 8.0 equiv) in DMF for 1.5 h. After coupling, the resin was washed with DMF (4×). The next Fmoc‐Lys(Cbz)‐OH was coupled by the same protocol. In the following cycle, a 2.5‐fold excess of Fmoc‐3‐aminomethyl‐Phac‐OH, HBTU, HOBt in presence of 5.0 equiv. DIPEA was coupled. After Fmoc deprotection and washing, the resin was dried *in vacuo* (2.53 g, loading 0.64 mmol/g).

200 mg of this resin were coupled with Fmoc‐Gly‐OH in a 2 mL polypropylene syringe, followed by Fmoc deprotection and washing with DMF (2×) and CH_2_Cl_2_ (4×). Subsequently, the peptide was removed from resin by treatment with 1 % TFA in CH_2_Cl_2_ (*v*/*v*; 4×30 min), the solution was immediately neutralized with DIPEA after each cleavage. The solvent was removed *in vacuo* providing the crude intermediate **34** (HPLC retention time 19.61 min, start at 30 % solvent B; MS calcd *m/z* 974.51, found *m/z* 975.44 [*M*+H]^+^).

Intermediate **35**. The crude intermediate **34** (≈125 μmol, calculated based on the original resin loading) was dissolved in 60 mL DMF (≈2 mmol/L). Over a period of 1 h, a solution of HATU (143 mg, 375 μmol, 3.0 equiv) in 5 mL DMF and a solution of DIPEA (131 μL, 750 μmol, 6.0 equiv) in 5 mL were added in equal amounts. The reaction mixture was stirred for further 2 h. The solvent was removed *in vacuo* and the obtained crude cyclic product was dissolved in 2.5 mL of 4.0 M HCl in dioxane. After 3 h, the reaction mixture was poured into cold Et_2_O. After centrifugation, the precipitate was purified by preparative HPLC (gradient: 30 % solvent B →90 % B in 60 min) yielding 35.8 mg (36.9 μmol) of compound **35** after lyophilization (HPLC retention time 16.73 min, start at 30 % solvent B; MS calcd *m/z* 856.46, found *m/z* 857.47 [*M*+H]^+^).

Inhibitor **10**. 35.8 mg (36.9 μmol, 1.0 equiv) of compound **35**, 51.5 mg (166 μmol, 4.5 equiv) *N,N’*‐di‐Boc‐1*H*‐pyrazole‐1‐carboxamidine, and 48.3 μL (277 μmol, 7.5 equiv) DIPEA were dissolved in 3 mL DMF and stirred for 48 h. The solvent was removed *in vacuo* and the remaining residue was treated with 2 mL of 33 % HBr in AcOH. After 2 h, the reaction mixture was poured into cold Et_2_O. After centrifugation, the precipitate was purified by preparative HPLC (0 % solvent B for 20 min, then 0 % solvent B→60 % solvent B in 120 min) providing 21.1 mg (21.7 μmol, 17 % based on the loaded Boc‐dLys(Fmoc)‐2‐CTC‐resin) of inhibitor **10** after lyophilization. (HPLC retention time 16.03 min, start at 1 % solvent B; MS calcd *m/z* 630.40, found *m/z* 631.44 [*M*+H]^+^).

**Enzyme kinetics**. Measurements with bZiPro^15^ and the covalent WNV NS2B‐NS3 construct^10^ were performed at room temperature in black 96 well plates using a Fluoroskan Ascent^®^ plate reader (Thermo Fisher Scientific, Vantaa, Finland) with *λ*_ex_ at 355 nm and *λ*_em_ at 460 nm. Each well contained 125 μL inhibitor dissolved in buffer (for bZiPro: 20 mM Tris×HCl pH 8.5, 2 mM DTT, 10 *w*/*v* glycerol and 0.01 *w*/*v* Triton X‐100;^18^ for WNV protease: 100 mM Tris×HCl pH 8.5, containing 32 *w*/*v* glycerol and 0.01 *w*/*v* Triton X‐100^12^) and 50 μL substrate Phac‐Leu‐Lys‐Lys‐Arg‐AMC^12^ (40 μM for bZiPro (10 μM in assay, *K*_M_=1.4 μM) and 400 μM for the WNV protease (100 μM in assay, *K*_M_=53 μM)). The measurements were started by addition of 25 μL enzyme solution (16 nM for bZiPro (2.0 nM in assay), and 64 nM for the WNV protease (8.0 nM in assay)). Data points were collected each 15 s over a period of 10 min using eight different inhibitor concentrations. The steady‐state rates in presence of inhibitor (*v*_i_) were calculated from the slopes of the progress curves and the (*v*_i_, [*I*]) data pairs were fitted to Equation (1) for a competitive reversible inhibition providing the *K*_i_ values. The *K*_i_ values and their standard deviation were obtained from at least three independent measurements.

(1) $$v_{i}=\frac{V_{\max}\times \left[S\right]}{K_{M}\left(1+\frac{\left[I\right]}{K_{i}}\right)+\left[S\right]}$$

The constants *K*_M_ and *V*_max_ were obtained from independent measurements with six different substrate concentrations in absence of inhibitor. For inhibitor **10** with bZiPro, additional measurements at three substrate concentrations (10, 5, and 2.5 μM in assay) were performed to obtain the Dixon plot shown in Figure 4.

Measurements with the dengue 4 virus protease construct (bD4Pro; expressed and purified by a similar methodology as described for bZiPro^15^ with slight modification of using Tobacco Etch Virus (TEV) protease instead of thrombin for His_6_‐tag cleavage) were performed in a Cytation 3 Mulitmode plate reader with *λ*_ex_ at 380 nm and *λ*_em_ at 460 nm. Assays were carried out as triplicate at 37 °C. Before measurement, the protease (final assay concentration 3 nM) was preincubated with varying concentrations of inhibitors in assay buffer (20 mM Tris pH 8.5, 10 % glycerol, 0.01 % Triton X‐100) for 1 h at room temperature in Corning black clear bottom 96‐well plates. The reaction was started by addition of the substrate Benzoyl‐Nle‐Lys‐Arg‐Arg‐AMC (final assay concentration 50 μM, *K*_M_=48.4 μM). Fluorescent readings were recorded at 1‐minute intervals over 10 minutes. The steady‐state rates *v*_i_ in presence of inhibitor were calculated from the slopes of the progress curves using Graph Pad Prism. The *v*_i_ values were fitted to Equation (2) as function of the inhibitor concentration [I] providing the IC_50_ values, where *v*_0_ is the rate in absence of inhibitor and *s* a slope factor. The obtained IC_50_ values were finally converted into *K*_i_ values by the Cheng‐Prusoff Equation (2).

(2) $$v_{i}\;=\frac{v_{0}}{{1+\left(\frac{\left[I\right]}{{IC}_{50}}\right)}^{s}}$$

(3) $$K_{i}\;=\frac{{\mathrm{IC}}_{50}}{\left(1+\frac{\left[S\right]}{K_{M}}\right)}$$

**Crystallization and structure determination of bZiPro**. The bZiPro/inhibitor complexes (molar ratio 1 : 3) were incubated for 1 h on ice at a protein concentration of 40 mg/mL. Different crystallization buffers were used for the individual complexes. 1 μL of the bZiPro/**2** or bZiPro/**9** mixture was mixed with 1 μL of reservoir solution (0.2 M ammonium sulfate, 0.1 M sodium acetate trihydrate pH 4.6, 25 % PEG 4000), 1 μL of the bZiPro/**4** or bZiPro/**8** mixture was mixed with 1 μL of reservoir solution (0.2 M ammonium sulfate, 0.1 M sodium acetate trihydrate pH 4.6, 30 % PEG 2000), 1 μL of the bZiPro/**10** or bZiPro/**15** mixture was mixed with 1 μL of reservoir solution (2 M ammonium sulfate, 0.1 M sodium acetate trihydrate pH 4.6), 1 μL of the bZiPro/**16** mixture was mixed with 1 μL of reservoir solution (2 M ammonium sulfate, 5 % propanol), and incubated at 18 °C in a hanging‐drop vapor diffusion experiment. Crystals appeared after two days and were cryoprotected using reservoir solution with 20 % glycerol before being flash‐cooled in liquid nitrogen.

Diffraction intensities of bZiPro/**4** complex were recorded at TPS 05A beamline at the National Synchrotron Radiation Research Center, Hsinchu, Taiwan. Diffraction intensities of bZiPro in complex with inhibitors **2**, **9**, and **16** were collected at MXII beamline at Australian Light Source, Melbourne, Australia. Diffraction intensities of bZiPro in complex with inhibitors **8** and **15** were collected at PSIII beamline at Swiss Light Source (SLS) Paul Scherrer Institut, Switzerland. Diffraction intensities of bZiPro in complex with inhibitor **10** were collected at BESSY MX beamline 14.1 at Helmholtz‐Zentrum Berlin, Germany.

Diffraction data for complexes with inhibitors **2**, **4**, **8**, **9**, **15** and **16** were indexed and integrated using iMOSFLM^38^ and scaled using Aimless.^39^, ^40^ Data processing and scaling for the complex with inhibitor **10** were performed with the XDS program package.^41^ The structure determinations for the bZiPro‐inhibitor complex crystal structures were done by molecular replacement with the program MOLREP and PHASER MR using the bZiPro structure as a model (PDB ID: 5GPI).^42^, ^43^ The structures were subjected to iterative rounds of refinement using the PHENIX^44^, ^45^ refine (5 cycles for inhibitor **10** and 3 cycles for the rest of the inhibitors) and manual rebuilding using Coot^46^ to fit amino acids into σ‐weighted 2*F*_o_−*F*_c_ and *F*_o_−*F*_c_ electron density maps. Simulated annealing was performed with Phenix to remove potential bias from the search model. TLS parameters were used subsequently in combination with isotropic *B*‐factor refinement, whereupon the TLS groups were automatically defined with Phenix. For the calculation of *R*_free_, 5 % of all data were randomly chosen and were not considered during refinement. Coordinates and restraints of inhibitor **10** were obtained from Grade Web server,^47^ whereas coordinates and restraints of inhibitor **2**, **4**, **8**, **9**, **15** and **16** were generated from PHENIX eLBOW.^44^ Multiple residue conformations, water molecules, ions, solvents, and inhibitors were located in the electron density and gradually added to the model. Multiple side‐chain conformations were maintained if the minor populated conformation displayed at least 20 % occupancy. Hydrogens were added in the penultimate refinement cycle, whereby the hydrogen atoms on the inhibitors were deleted in the last step. The final data collection and refinement statistics are summarized in Table S2. The structures were deposited in the Protein Data Bank. Figures were generated using PyMOL.^48^

**Accession codes**. PDB ID: 6KK2 (**2**), 6KK3 (**4**), 6KPQ (**8**), 6KK4 (**9**), 6Y3B (**10**), 6KK5 (**15**), and 6KK6 (**16**).

**Supporting information**. The analytical data of the synthesized inhibitors, a table with the data collection and refinement statistics of the bZiPro/inhibitor complexes, electron density maps of the bZiPro/inhibitors complexes, and results of stability tests for the incubation of bZiPro with selected inhibitors are provided as Supporting Information.

## Abbreviations

6‐Ahx: 6‐aminocaproic acid
5‐Ava: 5‐aminovaleric acid
Boc: *tert*‐butyloxycarbonyl
bD4Pro: unlinked binary dengue‐4 virus NS2B‐NS3 protease
bZiPro: unlinked binary Zika virus NS2B‐NS3 protease
Cbz: benzyloxycarbonyl
2‐CTC: 2‐chlorotrityl chloride
DIPEA: *N,N*‐diisopropylethylamine
DENV: dengue virus
DMF: *N,N*‐dimethylformamide
DTT: dithiothreitol
GABA: γ‐aminobutyric acid
HATU: 1‐[Bis(dimethylamino)methylene]‐1*H*‐1,2,3‐triazolo[4,5‐b]pyridinium 3‐oxide hexafluorophosphate
HBTU: 3‐[Bis(dimethylamino)methyliumyl]‐3*H*‐benzotriazol‐1‐oxide hexafluorophosphate
N(Ca)Lys: Nα(carbamidoyl)lysine
N(Ca)Orn: Nα(carbamidoyl)ornithine
Phac: phenylacetyl
SPPS: solid‐phase peptide synthesis
TFA: trifluoroacetic acid
WNV: West Nile virus
ZIKV: Zika virus

## Conflict of interest

The authors declare no conflict of interest.

## Supporting information

As a service to our authors and readers, this journal provides supporting information supplied by the authors. Such materials are peer reviewed and may be re‐organized for online delivery, but are not copy‐edited or typeset. Technical support issues arising from supporting information (other than missing files) should be addressed to the authors.

Supplementary

## References

[1] B. M. Kümmerer , S. M. Amberg , C. M. Rice in Handbook of Proteolytic Enzymes, 3rd ed. (Eds.: N. D. Rawlings, G. Salvesen), Academic Press, San Diego, 2013, pp. 3112–3120.

[2] C. Nitsche , Biophys. Rev. Lett. 2019, 11, 157–165. 10.1007/s12551-019-00508-3 PMC6441445 30806881

[3] S. A. Shiryaev , B. I. Ratnikov , A. E. Aleshin , I. A. Kozlov , N. A. Nelson , M. Lebl , J. W. Smith , R. C. Liddington , A. Y. Strongin , J. Virol. 2007, 81, 4501–4509. 17301157 10.1128/JVI.02719-06 PMC1900165

[4] S. Voss , C. Nitsche , Bioorg. Med. Chem. Lett. 2020, 30, 126965. 31980339 10.1016/j.bmcl.2020.126965

[5] Z. Yin , S. J. Patel , W. L. Wang , G. Wang , W. L. Chan , K. R. Rao , J. Alam , D. A. Jeyaraj , X. Ngew , V. Patel , D. Beer , S. P. Lim , S. G. Vasudevan , T. H. Keller , Bioorg. Med. Chem. Lett. 2006, 16, 36–39. 16246553 10.1016/j.bmcl.2005.09.062

[6] Z. Yin , S. J. Patel , W. L. Wang , W. L. Chan , K. R. Ranga Rao , G. Wang , X. Ngew , V. Patel , D. Beer , J. E. Knox , N. L. Ma , C. Ehrhardt , S. P. Lim , S. G. Vasudevan , T. H. Keller , Bioorg. Med. Chem. Lett. 2006, 16, 40–43. 16246563 10.1016/j.bmcl.2005.09.049

[7] P. Erbel , N. Schiering , A. D′Arcy , M. Renatus , M. Kroemer , S. P. Lim , Z. Yin , T. H. Keller , S. G. Vasudevan , U. Hommel , Nat. Struct. Mol. Biol. 2006, 13, 372–373. 16532006 10.1038/nsmb1073

[8] M. J. Stoermer , K. J. Chappell , S. Liebscher , C. M. Jensen , C. H. Gan , P. K. Gupta , W. J. Xu , P. R. Young , D. P. Fairlie , J. Med. Chem. 2008, 51, 5714–5721. 18729351 10.1021/jm800503y

[9] H. A. Lim , J. Joy , J. Hill , S. B. C. Chia , Eur. J. Med. Chem. 2011, 46, 3130–3134. 21565434 10.1016/j.ejmech.2011.04.055

[10] J. Lei , G. Hansen , C. Nitsche , C. D. Klein , L. Zhang , R. Hilgenfeld , Science 2016, 353, 503–505. 27386922 10.1126/science.aag2419

[11] C. Nitsche , L. Zhang , L. F. Weigel , J. Schilz , D. Graf , R. Bartenschlager , R. Hilgenfeld , C. D. Klein , J. Med. Chem. 2017, 60, 511–516. 27966962 10.1021/acs.jmedchem.6b01021

[12] M. Z. Hammamy , C. Haase , M. Hammami , R. Hilgenfeld , T. Steinmetzer , ChemMedChem 2013, 8, 231–241. 23307694 10.1002/cmdc.201200497

[13] G. Robin , K. Chappell , M. J. Stoermer , S. H. Hu , P. R. Young , D. P. Fairlie , J. L. Martin , J. Mol. Biol. 2009, 385, 1568–1577. 19059417 10.1016/j.jmb.2008.11.026

[14] C. G. Noble , C. C. Seh , A. T. Chao , P. Y. Shi , J. Virol. 2012, 86(1), 438–446. 22031935 10.1128/JVI.06225-11 PMC3255909

[15] Z. Zhang , Y. Li , Y. R. Loh , W. W. Phoo , A. W. Hung , C. Kang , D. Luo , Science 2016, 354, 1597–1600. 27940580 10.1126/science.aai9309

[16] Y. Li , Z. Zhang , W. W. Phoo , Y. R. Loh , W. Wang , S. Liu , M. W. Chen , A. W. Hung , T. H. Keller , D. Luo , C. Kang , Structure 2017, 25, 1242–1250. 28689970 10.1016/j.str.2017.06.006

[17] Y. Li , Z. Zhang , W. W. Phoo , Y. R. Loh , R. Li , H. Y. Yang , A. E. Jansson , J. Hill , T. H. Keller , K. Nacro , D. Luo , C. Kang , Structure 2018, 26, 555–564. 29526431 10.1016/j.str.2018.02.005

[18] W. W. Phoo , Z. Zhang , M. Wirawan , E. J. C. Chew , A. B. L. Chew , J. Kouretova , T. Steinmetzer , D. Luo , Antiviral Res. 2018, 160, 17–24. 30315877 10.1016/j.antiviral.2018.10.006

[19] C. Nitsche , H. Onagi , J. P. Quek , G. Otting , D. Luo , T. Huber , Org. Lett. 2019, 21, 4709–4712. 31188009 10.1021/acs.orglett.9b01545

[20] M. Dixon , Biochem. J. 1953, 55, 170–171. 13093635 10.1042/bj0550170 PMC1269152

[21] A. Thakkar , T. B. Trinh , D. Pei , ACS Comb. Sci. 2013, 15, 120–129. 23265659 10.1021/co300136j PMC3570731

[22] M. S. Bernatowicz , Y. Wu , G. R. Matsueda , Tetrahedron Lett. 1993, 34, 3389–3392.

[23] V. Štrukil , Beilstein J. Org. Chem. 2017, 13, 1828–1849. 28904627 10.3762/bjoc.13.178 PMC5588497

[24] C. Nitsche , S. Holloway , T. Schirmeister , C. D. Klein , Chem. Rev. 2014, 114, 11348–11381. 25268322 10.1021/cr500233q

[25] H. Wu , S. Bock , M. Snitko , T. Berger , T. Weidner , S. Holloway , M. Kanitz , W. E. Diederich , H. Steuber , C. Walter , D. Hofmann , B. Weissbrich , R. Spannaus , E. G. Acosta , R. Bartenschlager , B. Engels , T. Schirmeister , J. Bodem , Antimicrob. Agents Chemother. 2015, 59, 1100–1109. 25487800 10.1128/AAC.03543-14 PMC4335830

[26] B. Millies , F. von Hammerstein , A. Gellert , S. Hammerschmidt , F. Barthels , U. Goppel , M. Immerheiser , F. Elgner , N. Jung , M. Basic , C. Kersten , W. Kiefer , J. Bodem , E. Hildt , M. Windbergs , U. A. Hellmich , T. Schirmeister , J. Med. Chem. 2019, 62, 11359–11382. 31769670 10.1021/acs.jmedchem.9b01697

[27] Y. Yao , T. Huo , Y. L. Lin , S. Nie , F. Wu , Y. Hua , J. Wu , A. R. Kneubehl , M. B. Vogt , R. Rico-Hesse , Y. Song , J. Am. Chem. Soc. 2019, 141, 6832–6836. 31017399 10.1021/jacs.9b02505 PMC6501818

[28] A. Coluccia, M. Puxeddu, M. Nalli, C.-K. Wei, Y.-H. Wu, E. Mastrangelo, T. Elamin, D. Tarantino, J. J. Bugert, B. Schreiner, J. Nolte, F. Schwarze, G. La Regina, J.-C. Lee, R. Silvestri, *ACS Med. Chem. Lett* **2020**, *ASAP*, DOI: 10.1021/acsmedchemlett.9b00405. 10.1021/acsmedchemlett.9b00405 PMC7549096 33062166

[29] C. Nitsche , T. Passioura , P. Varava , M. C. Mahawaththa , M. M. Leuthold , C. D. Klein , H. Suga , G. Otting , ACS Med. Chem. Lett. 2019, 10, 168–174. 30783498 10.1021/acsmedchemlett.8b00535 PMC6378662

[30] H. A. Lim , M. J. Ang , J. Joy , A. Poulsen , W. Wu , S. C. Ching , J. Hill , C. S. Chia , Eur. J. Med. Chem. 2013, 62, 199–205. 23353753 10.1016/j.ejmech.2012.12.043

[31] N. Kurt Yilmaz , R. Swanstrom , C. A. Schiffer , Trends Microbiol. 2016, 24, 547–557. 27090931 10.1016/j.tim.2016.03.010 PMC4912444

[32] A. K. Ghosh , H. L. Osswald , G. Prato , J. Med. Chem. 2016, 59, 5172–5208. 26799988 10.1021/acs.jmedchem.5b01697 PMC5598487

[33] S. E. Allen , N. V. Dokholyan , A. A. Bowers , ACS Chem. Biol. 2016, 11, 10–24. 26575401 10.1021/acschembio.5b00663

[34] A. Tziridis , D. Rauh , P. Neumann , P. Kolenko , A. Menzel , U. Bräuer , C. Ursel , P. Steinmetzer , J. Stürzebecher , A. Schweinitz , T. Steinmetzer , M. T. Stubbs , Biol. Chem. 2014, 395, 891–903. 25003390 10.1515/hsz-2014-0158

[35] S. O. Dahms , M. Arciniega , T. Steinmetzer , R. Huber , M. E. Than , Proc. Natl. Acad. Sci. USA 2016, 113, 11196–11201. 27647913 10.1073/pnas.1613630113 PMC5056075

[36] J. Kouretova , M. Z. Hammamy , A. Epp , K. Hardes , S. Kallis , L. Zhang , R. Hilgenfeld , R. Bartenschlager , T. Steinmetzer , J. Enzyme Inhib. Med. Chem. 2017, 32, 712–721. 28385094 10.1080/14756366.2017.1306521 PMC6445162

[37] S. Pach , T. M. Sarter , R. Yousef , D. Schaller , S. Bergemann , C. Arkona , J. Rademann , C. Nitsche , G. Wolber , ACS Med. Chem. Lett. 2020, 11, 514–520. 32292558 10.1021/acsmedchemlett.9b00629 PMC7153273

[38] T. G. Battye , L. Kontogiannis , O. Johnson , H. R. Powell , A. G. Leslie , Acta Crystallogr. Sect. D 2011, 67, 271–281. 21460445 10.1107/S0907444910048675 PMC3069742

[39] P. R. Evans , Acta Crystallogr. Sect. D 2011, 67, 282–292. 21460446 10.1107/S090744491003982X PMC3069743

[40] P. R. Evans , G. N. Murshudov , Acta Crystallogr. Sect. D 2013, 69, 1204–1214. 23793146 10.1107/S0907444913000061 PMC3689523

[41] W. Kabsch , Acta Crystallogr. Sect. D 2010, 66, 133–144. 20124693 10.1107/S0907444909047374 PMC2815666

[42] A. J. McCoy , Acta Crystallogr. Sect. D 2007, 63, 32–41. 17164524 10.1107/S0907444906045975 PMC2483468

[43] A. Vagin , A. Teplyakov , Acta Crystallogr. Sect. D 2010, 66, 22–25. 20057045 10.1107/S0907444909042589

[44] P. D. Adams , P. V. Afonine , G. Bunkoczi , V. B. Chen , I. W. Davis , N. Echols , J. J. Headd , L. W. Hung , G. J. Kapral , R. W. Grosse-Kunstleve , A. J. McCoy , N. W. Moriarty , R. Oeffner , R. J. Read , D. C. Richardson , J. S. Richardson , T. C. Terwilliger , P. H. Zwart , Acta Crystallogr. Sect. D 2010, 66, 213–221. 20124702 10.1107/S0907444909052925 PMC2815670

[45] P. V. Afonine , R. W. Grosse-Kunstleve , N. Echols , J. J. Headd , N. W. Moriarty , M. Mustyakimov , T. C. Terwilliger , A. Urzhumtsev , P. H. Zwart , P. D. Adams , Acta Crystallogr. Sect. D 2012, 68, 352–367. 22505256 10.1107/S0907444912001308 PMC3322595

[46] P. Emsley , K. Cowtan , Acta Crystallogr. Sect. D 2004, 60, 2126–2132. 15572765 10.1107/S0907444904019158

[47] O. Smart, T. Womack, Grade Web Server, Vol. May 22, 2019.

[48] *The PyMOL Molecular Graphics System*, version 1.3; Schrödinger LLC: New York.

