# Supplementary material for: Structure‐Based Macrocyclization of Substrate Analogue NS2B‐NS3 Protease Inhibitors of Zika, West Nile and Dengue viruses
Source: ChemMedChem. 2020 Jun 30;15(15):1439–52. doi: 10.1002/cmdc.202000237 (PMC7497253; doi:10.1002/cmdc.202000237)
Supplement: Supplementary file 1 — Supplementary [file CMDC-15-1439-s001.pdf]

ChemMedChem

Supporting Information

Structure-Based Macrocyclization of Substrate Analogue NS2B-NS3 Protease Inhibitors of Zika, West Nile and Dengue viruses

Niklas J. Braun+, Jun P. Quek+, Simon Huber+, Jenny Kouretova, Dorothee Rogge, Heike Lang-Henkel, Ezekiel Z. K. Cheong, Bing L. A. Chew, Andreas Heine, Dahai Luo,* and Torsten Steinmetzer*

Content

1.	General information	S2
2.	Analytical data of final inhibitors	S3
3.	Structure determination of bZiPro in complex with selected inhibitors	S4
4.	Structures of bZiPro in complex with inhibitors **2**, **4**, **8**, **9**, **15**, and **16**	S6
5.	Backbone interactions of inhibitor **10** in complex with bZiPro	S7
6.	Stability test of selected inhibitors against degradation by bZiPro	S7
7.	References	S9

1. General Information

Analytical HPLC

Analytical HPLC experiments were performed on a Primaide (VWR, Hitachi) system (column, NUCLEODUR C18 ec, 5 µm, 100 Å, 4.6 mm × 250 mm, Macherey-Nagel, Düren, Germany). Water (solvent A) and acetonitrile (solvent B), both containing 0.1 % TFA, were used as eluents with a linear gradient (increase of 1 % solvent B/min, start at 1 % solvent B) and a flow rate of 1 mL/min. The detection was performed at 220 nm. All final inhibitors were lyophilized and obtained as TFA salts with a purity > 95 %, as determined by analytical HPLC detection at 220 nm.

Mass spectrometry

The molecular mass of the synthesized compounds was determined using a QTrap 2000 ESI spectrometer (Applied Biosystems).

2. Analytical data of final inhibitors

Table S1. Analytical data of the synthesized inhibitors.

No.	calcd *m/z*	found *m/z* $(M+H)^+$	HPLC retention time, start at 1% B (min)
**2**	658.43	659.48	17.83
**3**	644.41	645.46	17.68
**4**	658.85	659.49	18.17
**5**	644.41	645.49	17.79
**6**	616.81	617.48	17.31
**7**	602.78	603.50	17.07
**8**	658.43	659.38	18.68
**9**	644.41	645.41	17.76
**10**	630.40	631.44	17.03
**11**	616.38	617.55	16.53
**12**	573.38	574.41	17.95
**13**	630.40	631.38	17.59
**14**	644.41	645.29	18.32
**15**	644.41	645.64	18.63
**16**	644.41	645.61	18.01
**17**	672.44	673.40	21.95
**18**	672.44	673.50	21.63
**19**	670.43	671.51	20.51
**20**[a]	588.37	589.42	15.88
**21**[b]	631.38	632.25	16.71
**22**	568.38	569.33	12.51
**23**	582.40	583.41	13.20
**24**	596.41	597.36	14.53
**25**	597.37	598.40	12.00
**26**	611.39	612.42	11.89
**27**	611.39	612.70	12.56
**28**	611.39	612.45	11.77
**29**	648.41	649.49	14.48
**30**	647.42	648.53	14.45
**31**	632.41	633.68	16.60
**32**	589.41	590.46	18.24

[a] Structure of inhibitor **20**

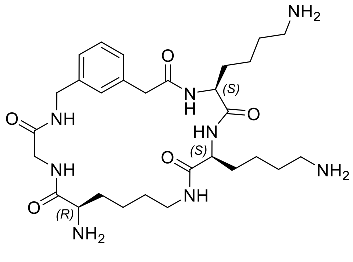

[b] Structure of inhibitor **21**

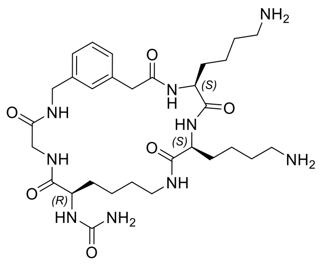

3. Structure determination of bZiPro in complex with inhibitors 2, 4, 8, 9, 10, 15, and 16

Table S2. Data collection and refinement statistics.

**Data Collection and Processing**<br>**PDB-ID**	**2**<br>6KK2	**4**<br>6KK3	**8**<br>6KPQ	**9**<br>6KK4	**10**<br>6Y3B	**15**<br>6KK5	**16**<br>6KK6
Wavelength (Å)	0.95372	1	0.97924	0.95373	0.9184	0.97924	0.95373
Beamline	ALS, MXII	TPS, 05A	SLS, PSIII	ALS, MXII	BESSY, MX 14.1	SLS, PSIII	ALS, MXII
Detector	ADSC Quantum 315r Detector	MX300HS	PILATUS 2M-F	ADSC Quantum 315r Detector	PILATUS 6M	PILATUS 2M-F	ADSC Quantum 315r Detector
Space group	$P\ 4_3\ 2\ 2$	$P\ 4_3\ 2\ 2$	$P\ 4_3\ 2\ 2$	$P\ 2_1\ 2_1\ 2_1$	$P\ 2_1\ 2_1\ 2_1$	$P\ 4_3\ 2\ 2$	$P\ 2_1\ 2_1\ 2_1$
Unit cell a, b, c (Å)	42.4 42.4 215.7	42.6 42.6 215.3	42.6 42.6 214.2	48.8 60.4 83.2	48.6 60.5 83.1	42.5 42.5 215.4	48.6 60.7 83.4
Matthews coefficient ($Å^3$/Da)	2.2	2.3	2.2	2.7	2.5	2.2	2.9
Solvent content (%)	44	45	45	55	50	45	57
**Diffraction Data**							
Resolution range (Å)	42.4 - 2.02 (2.10 - 2.02)	53.8 - 2.05 (2.12 - 2.05)	42.6 - 2.62 (2.71 - 2.62)	41.6 - 1.74 (1.80 - 1.74)	48.9 - 1.59 (1.69 - 1.59)	42.5 - 2.03 (2.10 - 2.03)	42.0 - 1.74 (1.80 - 1.74)
Total reflections	189875 (16956)	160734 (16002)	85505 (8791)	289548 (27245)	197383 (32248)	170433 (16674)	290724 (27264)
Unique reflections	13825 (1265)	13375 (1289)	6571 (629)	25939 (2483)	33699 (5398)	13722 (1317)	25997 (2478)
Multiplicity	13.7 (12.8)	12.0 (12.4)	13.0 (14.0)	11.2 (11.0)	5.9 (6.0)	12.4 (12.7)	11.2 (11.0)
Completeness (%)	99.4 (95.2)	99.9 (100.0)	99.8 (99.8)	99.7 (97.4)	99.8 (99.7)	99.8 (99.5)	99.6 (96.2)
Mean I/sigma (I)	35.1 (3.0)	10.6 (1.8)	12.1 (1.8)	36.0 (7.3)	21.2 (3.1)	18.5 (1.8)	21.6 (2.8)
Wilson B-factor ($Å^2$)	39.7	32.0	52.3	20.8	21.1	39.4	24.2
$^aR_{merge}$	0.196 (0.593)	0.155 (1.36)	0.192 (1.64)	0.044 (0.336)	0.043 (0.484)	0.091 (1.37)	0.073 (0.912)
$CC_{1/2}$	0.99 (0.96)	1.00 (0.72)	1.00 (0.84)	1.00 (0.96)	1.00 (0.86)	1.0 (0.86)	1.00 (0.83)
**Refinement statistics**							
Resolution range (Å)	42.4 - 2.02 (2.09 - 2.02)	53.8 - 2.05 (2.12 - 2.05)	42.56 - 2.62 (2.71 - 2.62)	41.6 - 1.74 (1.80 - 1.74)	48.9 - 1.59 (1.64 - 1.59)	42.5 - 2.03 (2.10 - 2.03)	42.0 - 1.74 (1.80 - 1.74)
Reflections work	13136 (1204)	12702 (1223)	6225 (595)	24642 (2344)	32014 (3146)	13036 (1249)	24698 (2357)
Reflections free	689 (61)	673 (66)	346 (34)	1297 (123)	1685 (165)	686 (68)	1299 (121)
$^bR_{work}$	0.219 (0.235)	0.189 (0.257)	0.218 (0.313)	0.162 (0.212)	0.162 (0.203)	0.198 (0.274)	0.172 (0.225)
$^cR_{free}$	0.256 (0.298)	0.217 (0.351)	0.246 (0.309)	0.184 (0.243)	0.188 (0.263)	0.231 (0.308)	0.181 (0.255)
Protein residues (NS2B/NS3)	39 / 156	38 / 154	38 / 153	39/ 161	38 / 159	38 / 153	38 / 152

Inhibitor atoms	47	47	47	46	45	46	46
Water molecules	38	76	4	143	129	51	99
Other ligand atoms	0	0	0	4	11	0	11
[d]RMSD (bonds) (Å)	0.008	0.015	0.009	0.017	0.007	0.008	0.007
RMSD (angles) (°)	1.23	1.66	1.38	1.86	0.92	1.25	1.29
[e]Ramachandran favored (%)	96.8	97.3	95.7	96.9	96.4	96.3	97.3
[e]Ramachandran allowed (%)	3.2	2.7	4.3	3.1	3.6	3.7	2.7
[e]Ramachandran outliers (%)	0.0	0.0	0.0	0.0	0.0	0.0	0.0
[f]Average B-factor ($Å^2$)	49.7	36.9	62.8	27.5	30.0	46.9	32.7
Protein	49.2	36.0	62.7	26.5	29.5	46.7	32.1
Inhibitor	65.2	51.2	64.4	18.3	20.0	49.2	22.5
Water molecules	49.2	44.6	53.2	40.0	39.0	50.2	43.2
Other ligands	-	-	-	38.8	40.8	-	58.2

Statistics for the highest-resolution shell are shown in parentheses.

[a]$R_{merge} = \sum|I_j - < I >|/\sum I_j$, where I_j is the intensity of an individual reflection, and $< I >$ is the average intensity of that reflection.

[b]$R_{work} = \sum||F_{obs}| - |F_{calc}||/\sum|F_{obs}|$, where F_{obs} denotes the observed structure factor amplitude, and F_c the structure factor amplitude calculated from the model.

[c]R_{free} is as for R_{work} but calculated with 5% of randomly chosen reflections omitted from the refinement.

[d]RMSD, root mean square deviations.

[e]Ramachandran, calculated using MOLPROBITY[1]

[f]Average B-factor, calculated using MOLEMAN[2-3]

4. Structures of bZiPro in complex with inhibitors 2, 4, 8, 9, 15, and 16

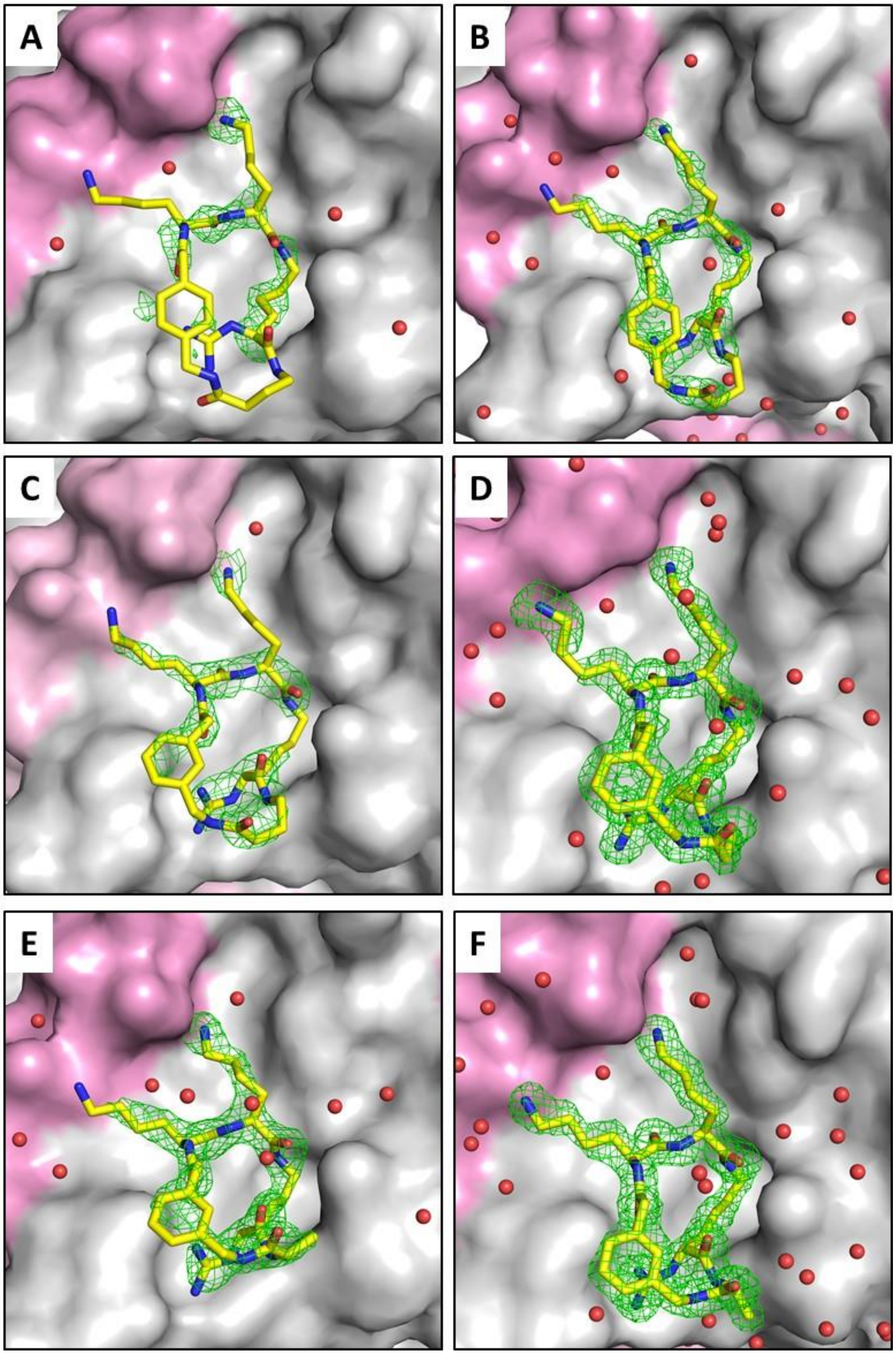

Figure S1. Electron density maps of the inhibitors **2** (A), **4** (B), **8** (C), **9** (D), **15** (E) and **16** (F) in the determined complexes with bZiPro (given in surface representation, NS2B in pink, NS3 in gray). Inhibitors are shown as stick model with carbon atoms in yellow. The $\mathbf{F_o}$-$\mathbf{F_c}$ omit-electron density maps (green mesh) are contoured at 2.5σ for inhibitor **2** and **8**, contoured at 3σ for inhibitor **4, 9, 15** and **16**.

5. Structures of bZiPro in complex with inhibitors 10 indicating the backbone interactions of the inhibitor

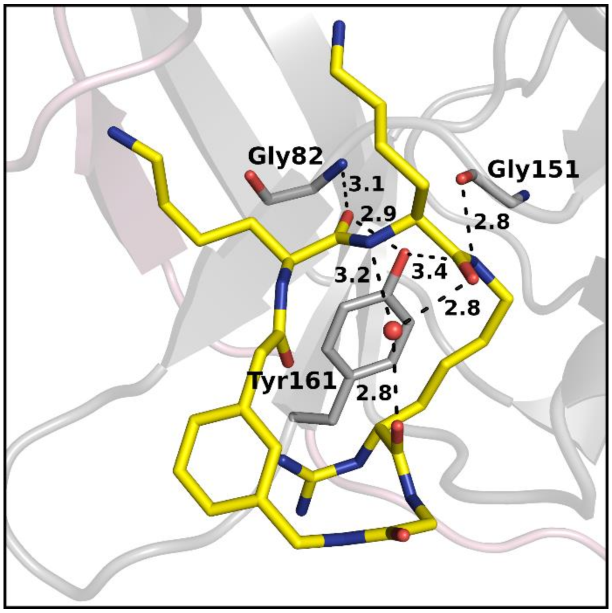

Figure S2. Backbone interactions of inhibitor **10** in complex with bZiPro.

6. Stability test of selected inhibitors against degradation by bZiPro.

The inhibitor stability assay was carried out as previously described[4]. The inhibitors, **2**, **4**, **9**, **10**, **15** and **16**, were incubated with bZiPro at 10:1 ratio (inhibitor: bZiPro) for 4 h at 37 °C. The same concentration of each inhibitor in the absence of bZiPro was prepared as negative controls. Analytical reverse phase chromatography was performed using ACQUITY UPLC BEH C18 column, 2.1×100 mm (Waters, USA), thermostated at 65 °C, with an Acquity H-Class UPLC coupled to UPLC PDA and QDa Mass Detector (Waters, USA), with Mobile phase A in ultrapure water containing 0.1 % formic acid and mobile phase B in methanol containing 0.1 % formic acid. The mobile phase was delivered at a flow rate of 0.4 mL/min with the following gradient: 0–2 min, 10 % B; 2–4 min, 80 % B; 4–6 min, 80 % B; followed by a return to 10 % B at 9 min. Absorption was measured at 280 nm and 260 nm. The QDa ESI source was operated in positive mode, capillary voltage of 0.8 V, cone voltage of 10 V, probe temperature at 600 °C, over a

scan range of 150–1250 m/z at a frequency of 8 Hz. The chromatograms and mass spectra were acquired and analysed using Waters Masslynx 4.1 and deconvoluted using MaxEnt1.

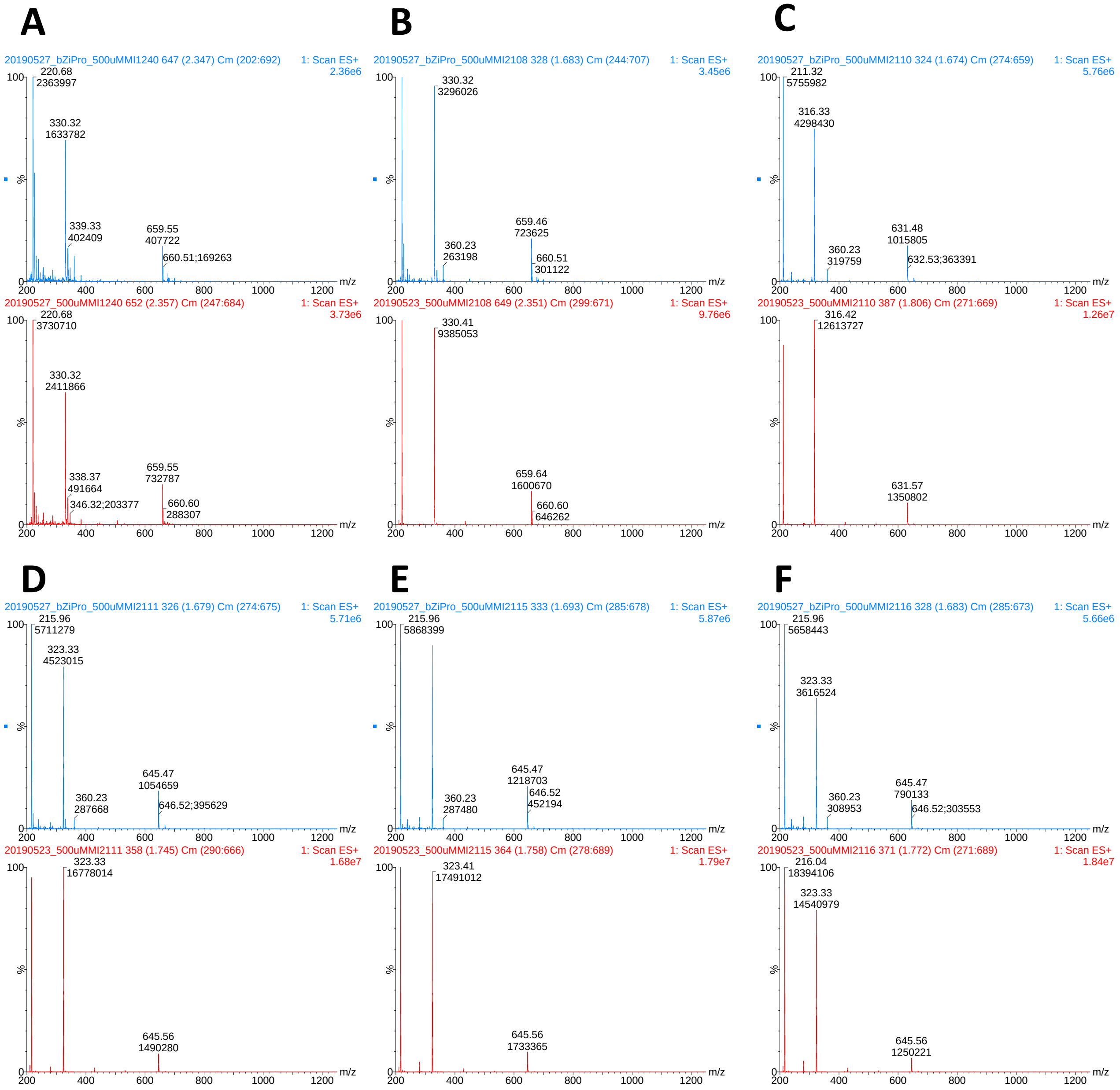

Figure S3. Mass spectra of the compounds before and after incubation with bZiPro determined by ESI-MS. In contrast to our previously described linear inhibitors[4], all cyclic compounds are stable against bZiPro-catalyzed cleavage. The top panel represent the mass spectra after incubation with bZiPro, while the bottom panel show the mass spectra in the absence of bZiPro. (A) Mass spectra of inhibitor **2**, (B) inhibitor **4**, (C) inhibitor **10**, (D) inhibitor **9**, (E) inhibitor **15**, and (F) inhibitor **16**.

7. References

[1] C. J. Williams, J. J. Headd, N. W. Moriarty, M. G. Prisant, L. L. Videau, L. N. Deis, V. Verma, D. A. Keedy, B. J. Hintze, V. B. Chen, S. Jain, S. M. Lewis, W. B. Arendall, 3rd, J. Snoeyink, P. D. Adams, S. C. Lovell, J. S. Richardson, D. C. Richardson, *Protein Sci.* **2018**, *27*, 293-315.

[2] G. J. Kleywegt, MOLEMAN, Uppsala University: Uppsala, Sweden.

[3] G. J. Kleywegt, J. Y. Zou, M. Kjeldgaard, T. A. Jones, Around O, in In International Tables for Crystallography (Eds.: M. G. Rossmann, E. Arnold), Kluwer Academic Publishers, Dordrecht, **2001**, pp. 353-356.

[4] W. W. Phoo, Z. Zhang, M. Wirawan, E. J. C. Chew, A. B. L. Chew, J. Kouretova, T. Steinmetzer, D. Luo, *Antiviral Res.* **2018**, *160*, 17-24.
